# Safety and efficacy of different antibiotic regimens in patients with ocular toxoplasmosis: systematic review and meta-analysis

**DOI:** 10.1186/s13643-021-01758-7

**Published:** 2021-07-19

**Authors:** John E. Feliciano-Alfonso, Juliana Muñoz-Ortiz, María Alejandra Marín-Noriega, Andrés Vargas-Villanueva, Laura Triviño-Blanco, Natalia Carvajal-Saiz, Alejandra de-la-Torre

**Affiliations:** 1 Departamento de Medicina Interna, Facultad de Medicina, Universidad Nacional de Colombia, Bogotá, Colombia; 2 NeURos Research Group, Escuela de Medicina Y Ciencias de La Salud, Universidad del Rosario, Carrera 24 # 63 C 69, Bogotá, Colombia; 3 Escuela Superior de Oftalmología-Instituto Barraquer de América, Bogotá, Colombia

**Keywords:** Toxoplasmosis, ocular, Toxoplasma gondii, Uveitis, Anti-bacterial agents, Therapeutics

## Abstract

**Background:**

Ocular toxoplasmosis (OT) is the most common cause of posterior uveitis, which leads to visual impairment in a large proportion of patients. Antibiotics and corticosteroids lower the risk of permanent visual loss by controlling infection and inflammation. However, there remains disagreement regarding optimal antibiotic therapy for OT. Therefore, this systematic review and meta-analysis were performed to determine the effects and safety of existing antibiotic treatment regimens for OT.

**Methods:**

MEDLINE, EMBASE, The Cochrane Central Register of Controlled Trials, LILACS, WHO International Clinical Trials Registry Platform portal, ClinicalTrials.gov, and Gray Literature in Europe (“OpenGrey”) were searched for relevant studies; manual searches of reference lists were performed for studies identified by other methods. All published and unpublished randomized controlled trials that compared antibiotic schemes known to be effective in OT at any dosage, duration, and administration route were included. Studies comparing antibiotics with placebo were excluded. This review followed standard methodological procedures recommended by the Cochrane group.

**Results:**

Ten studies were included in the narrative summary, of which four were included for quantitative synthesis (meta-analysis). Interventions were organized into three groups: intravitreal clindamycin versus pyrimethamine + sulfadiazine, trimethoprim + sulfamethoxazole versus other antibiotics, and other interventions. The first comparison favored intravitreal clindamycin (Mean difference (MD) = 0.10 logMAR; 95% confidence interval = 0.01 to 0.22). However, this finding lacks clinical relevance. Other outcomes showed no statistically significant differences between the treatment groups. In general, the risk of performance bias was high in evaluated studies, and the quality of the evidence found was low to very low.

**Conclusions:**

No antibiotic scheme was superior to others, and the selection of a treatment regimen depends on multiple factors; therefore, treatment should be chosen based on safety, sulfa allergies, and availability.

**Supplementary Information:**

The online version contains supplementary material available at 10.1186/s13643-021-01758-7.

## Background

Ocular toxoplasmosis (OT) is a primary cause of posterior uveitis worldwide, particularly in South America [1, 2]. OT results from an acquired or congenital infection by the obligate intracellular protozoan parasite *Toxoplasma gondii (Tg),* which can infect both humans and animals. An estimated 25%–30% of the human population is infected with *Tg* [3]. Seroprevalence is low (approximately 10%–30%) in Southeast Asia, Northern Europe, and North America. In Central and Southern Europe, the prevalence reported varies between 30 and 50%. Latin America and some tropical countries in Africa have higher seroprevalence (up to 80%) [4].

*Tg* infection in humans can be congenital or postnatally acquired through contaminated hands, food, water, consumption of undercooked and raw meat, organ transplantation, blood transfusion, or vertical transmission [5–7]. Toxoplasmosis has a variety of clinical manifestations in humans. Congenital toxoplasmosis is associated with intrauterine growth restriction, neurological, mental disease, hydrocephalus, encephalitis, retinochoroiditis, hearing and cardiovascular abnormalities, and fetal loss; it may also be asymptomatic [8, 9]. Acquired infection is asymptomatic in more than 80% of immunocompetent patients. In contrast, 20% of infected individuals may experience ocular compromise, fever, or cervical lymphadenopathy; these manifestations are occasionally associated with myalgia, asthenia, or other nonspecific clinical signs [10]. The most common manifestation of OT is toxoplasmic retinochoroiditis, which typically comprises a unilateral, unifocal, necrotizing retinochoroidal lesion that appears as a whitish-yellow region with a blurred margin, with or without an accompanying old lesion (often associated with vitritis) [11].

The treatment goals in OT are reducing the risk of visual impairment by avoiding the multiplication of the parasite during the active stage of retinochoroiditis, and diminishing the necrotizing inflammation and subsequent harm to adjacent tissues [12]. In addition, because antibiotic treatment is only effective against the tachyzoite form of the parasite, it is important to treat each active lesion, which presumably reduces the risk of recurrence [12, 13].

Antibiotic therapy in acute lesions of OT is highly recommended for the reduction of intraocular inflammation and the prevention of retinal and optic disc damage. Unfortunately, current treatment schemes do not eradicate *Tg* tissue cysts; thus, they do not prevent further reactivation of toxoplasma retinochoroiditis unless prophylaxis is started [14].

Various treatment regimens are often used in clinical practice. The classic triple therapy is pyrimethamine + sulfadiazine (PYR/SDZ) combined with folinic acid, which is the first choice for congenital toxoplasmosis and immunosuppressed patients; PYR/SDZ + clindamycin + folinic acid is known as quadruple therapy; trimethoprim + sulfamethoxazole (TMP/SMX) can be used alone or combined with clindamycin; for individuals allergic to sulfa, azithromycin + pyrimethamine + folinic acid or clindamycin + pyrimethamine + folinic acid can be used; and clindamycin alone can be used for intravitreal therapy [14]. In addition, corticosteroids can be used as an adjuvant treatment. Oral prednisolone at 1 mg/kg/day is administered from the third day until 2–6 weeks of treatment. Intravitreal therapy also includes clindamycin combined with dexamethasone [15].

In survey studies, ophthalmologists were asked about the therapies they used to manage active OT; the results revealed a diverse range of treatment regimens, and many ophthalmologists expressed doubtfulness about questions regarding the disease. The diversity of responses suggests widespread uncertainty regarding appropriate treatment for this disease; moreover, there is a need for continuing medical education regarding OT [16]. There have been some publications concerning this topic [13, 17, 18]. Notably, a systematic review of the literature was published in 2003 regarding antibiotics for toxoplasmic retinochoroiditis; however, it compared antibiotic schemes versus placebo and concluded that there was a lack of evidence to support routine antibiotic treatment [18]. Another systematic review compared antibiotic treatment versus placebo in patients with OT; it found a lack of evidence to support routine antibiotic treatment for toxoplasma retinochoroiditis to prevent visual impairment and found weak evidence regarding the prevention of recurrences. Thus, the authors of that study concluded that the risk of recurrence is likely reduced after long‐term treatment with systemic antibiotics [13]. Nonetheless, in ophthalmological clinical practice, antibiotic treatment must be provided to achieve treatment objectives for patients with OT. Our systematic review and meta-analysis will complement a recently published meta-analysis regarding treatment in immunocompetent patients [19].

This systematic review and meta-analysis aimed to evaluate the evidence-based information regarding the safety and efficacy of different existing antibiotic regimens in patients with OT worldwide.

## Methods

### Protocol and registration

The study protocol was developed based on the Preferred Reporting Items for Systematic Reviews and Meta-Analysis Protocols (PRISMA-P) guidelines [20], and it was previously published in 2019 [21]. In the same way, it was registered in PROSPERO, with the registration number CRD42018085468 [22].

### Eligibility criteria

We included all published and unpublished randomized controlled trials (RCTs) comparing therapies that have been used in OT. RCTs provide the highest quality of evidence according to the grading of recommendation assessment, development, and evaluation (GRADE) classification [23, 24]. Therapies described in the literature for OT include trimethoprim-sulfamethoxazole, pyrimethamine, sulfadoxine, sulfadiazine, clindamycin, tetracyclines, clarithromycin, azithromycin, atovaquone, minocycline, spiramycin, rifabutin, trimetrexate, lincomycin, dapsone, sulfafurazole, ciprofloxacin, doxycycline, miokamycin, erythromycin, macrolide, sulfonamide, sulfamerazine, nifurtimox, methotrexate, alone or in combination [13, 25–28]. We also included therapies at any dosage, duration, and administration route, oral or intravitreal. RCTs comparing antibiotics with placebo were excluded because a systematic review of this comparison has already been conducted by Pradhan et al. [13]. Participants included were patients of any age who received antibiotic treatment for acute OT worldwide, and those with healed scars who received prophylactic antibiotic treatment to prevent recurrent or new lesions, including immunocompetent patients, immunosuppressed patients, pregnant women, and children.

### Outcomes

Primary outcome measures were changes in visual acuity at least three months after the start of treatment and the number of recurrences at the end of follow-up. Secondary outcome measures included the behavior of ocular inflammation signs according to the Standardization of Uveitis Nomenclature (SUN), size of lesion at the end of the follow-up, adverse drug reactions, and duration of the active lesion, as stated in the study protocol [21].

### Information sources

We used a combination of exploded controlled vocabulary with thesaurus *Science Health Descriptors (DeCS for its Spanish acronym), Medical Subject Heading (*MeSH) and Embase Subject Headings (Emtree), and free-text terms (considering spelling variants, plurals, synonyms, acronyms, and abbreviations) with field labels, truncation, proximity operators, and Boolean operators. We conducted our search in the MEDLINE, EMBASE, Cochrane Central Register of Controlled Trials, and LILACS electronic databases, from inception to March 2018 (Annex 1). Besides, the search was updated in November 2020. For identification of additional studies, the following resources were used: WHO International Clinical Trials Registry Platform portal, ClinicalTrials.gov, Gray Literature in Europe (“OpenGrey”), and manual searches within reference lists of all relevant studies identified by other methods.

### Study selection

Studies obtained by electronic databases were independently reviewed by two authors (JFA and AVV), evaluating titles and abstracts of all studies. Next, they independently compared the full text with the inclusion criteria. Finally, disagreements were resolved by consensus or by a third reviewer (ADLT).

### Data collection process

A data collection form was designed. Then, two review authors (JMO and JFA) independently extracted relevant details regarding the design and results of each study. Disagreements were resolved by consensus or by independent evaluation by a third review author (ADLT). One review author (JFA) entered data into Review Manager 5.3 (RevMan 5.3) software and a third reviewer (ADLT) checked them to ensure data quality.

### Risk of bias in individual studies

We used the Cochrane group “Risk of Bias” tool for RCTs and criteria in the Cochrane Handbook for Systematic Reviews of Interventions to assess these risks in the relevant domains of the reported methods and results from each included study. The five domains are selection bias, performance bias, attrition bias, detection bias, reporting bias, and other biases. These domains can be classified as high, unclear, and low risk of bias [29].

### Dichotomous data

We presented the results as risk ratios (RRs) with 95% confidence intervals (CIs) for dichotomous data.

### Continuous data

We used the mean difference for continuous data if outcomes were measured in the same manner among multiple trials. We used the standardized mean difference to combine trials that measured the same outcome using different methods.

### Management of missing data

We contacted study investigators to obtain missing data. We analyzed available data without making assumptions or imputing data to adjust for missing data.

### Assessment of heterogeneity

We assessed statistical heterogeneity in each meta-analysis using the I^2^ statistic. We regarded heterogeneity as substantial if I^2^ was > 40% and < 70%, which required using a random-effects model in the analysis. If there was substantial heterogeneity and I^2^ was > 70%, meta-analysis was not performed; instead, a narrative summary of data was conducted.

### Assessment of reporting biases

We used funnel plots to assess publication bias when at least 10 studies were available for meta-analysis. In addition, we evaluated possible sources of asymmetry in funnel plots, following the method described in the Cochrane Handbook for Systematic Reviews of Interventions [29].

### Data synthesis

We used a random-effects model to calculate our meta-analysis if eligible studies represented clinically varied populations. In addition, we used a fixed-effect meta-analysis for combining data where it was reasonable to assume that studies estimated the same underlying treatment effect. Statistical analysis using RevMan 5.3 and GRADE assessment (Annex 2) was performed by one author (JFA). We assessed statistical heterogeneity in each meta-analysis using the I^2^ statistic. Additional information about the methodology is presented in the published protocol [21].

## Results

### Literature search results

**Fig. 1:**
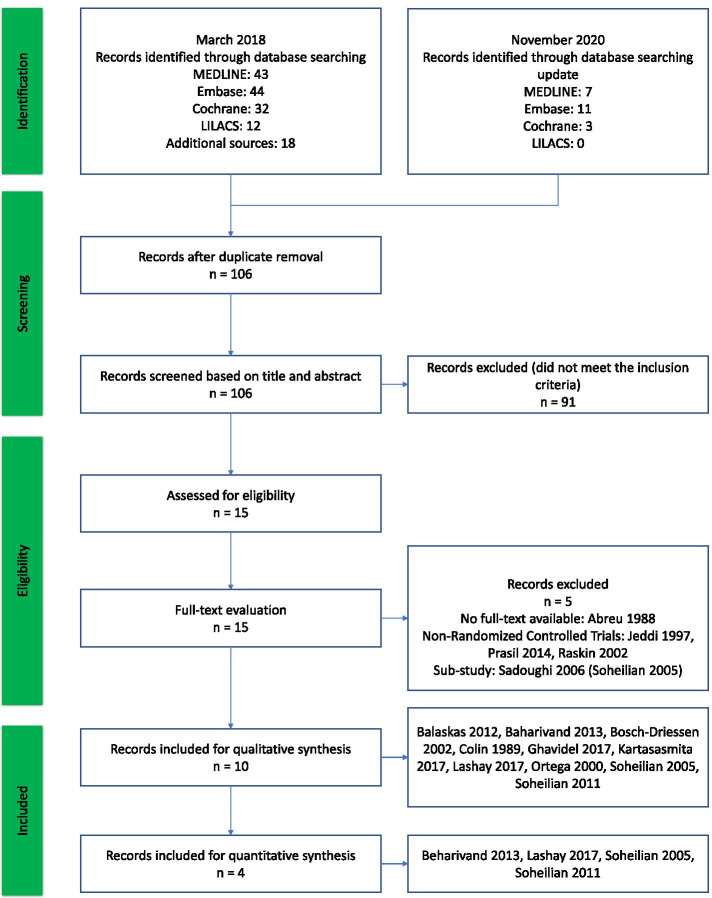
Study flow diagram

In total, 131 studies were identified in the electronic databases (44 in Embase, 43 in MEDLINE, 32 in Cochrane Central Register of Controlled Trials, and 12 in LILACS); 18 references were identified in the additional sources. After duplicates had been removed, 94 records were screened; 15 were selected for full-text evaluation. Five studies were excluded for reasons indicated in Fig. 1 [30–35]. In the case of Sadoughi et al. 2006 study [35], the same population was also evaluated by Soheilian 2005; thus, we choose the study with a longer follow-up, which corresponded to Soheilian 2005 [30].

The remaining 10 studies were included for narrative summary of data (Balaskas 2012 [36], Baharivand 2013 [37], Bosch-Driessen 2002 [38], Colin 1989 [39], Ghavidel 2017 [40], Kartasasmita 2017 [41], Lashay 2017 [42], Ortega 2000 [43], Soheilian 2005 [30], Soheilian 2011 [44]); four provided sufficient information for quantitative synthesis (meta-analysis) (Baharivand 2013 [37], Lashay 2017 [42], Soheilian 2005 [30], Soheilian 2011 [44]).

In the update 2020, 21 new studies were identified in the electronic databases (11 in Embase, 7 in MEDLINE, 3 in Cochrane Central Register of Controlled Trials, and 0 in LILACS). After duplicates had been removed, 12 records were screened. None of the studies evaluated in the title and abstract phase met the selection criteria.

### Characteristics of included studies

Most of the 10 included studies were conducted in Asia, particularly in Iran (Baharivand 2013 [37], Ghavidel 2017 [40], Lashay 2017 [42], Soheilian 2005 [30], Soheilian 2011 [44]) and Indonesia (Kartasasmita 2017 [41]). The remaining studies were conducted in Europe (Balaskas 2012 [36], Bosch-Driessen 2002 [38], Colin 1989 [39]) and North America (Ortega 2000 [43]). All 10 studies were published between 1989 and 2017. Eight studies used the classic therapy PYR/SDZ as a comparison group. Three studies included patients under 18 years of age (Colin 1989 [39], Ortega 2000 [43], Soheilian 2005 [30]); patient age ranged between 7 and 72 years in all included studies. In general, the studies had a small sample size, from 19 patients (Balaskas 2012 [36]) to 72 patients (Ghavidel 2017 [40]). A summary of all included studies can be found in Table 1, and Annex 3 provides detailed information regarding the 10 included studies.

**Table 1:** asas

Author, year	Location	Participants	Intervention Treatment	Control Treatment	Outcomes	Intervention	Control
**Balaskas, 2012**	Switzerland	*N* = 19 Intervention = 10 Control = 9	Azithromycin + prednisone Administration route: oral	PYR/SDZ + folinic acid + prednisone Administration route: oral	Changes in VA Number of recurrences Improvement in VA Ocular inflammation Retinal lesion Size	NR NR NR NR NR	NR NR NR NR NR
					Adverse drug reactions (minor)	NR	9 (100%)
**Baharivand, 2013**	Iran	*N* = 66 Intervention = 32 Control = 34	Clindamycin + dexamethasone Administration route: intravitreal	PYR/SDZ + folinic acid + prednisone Administration route: oral	Changes in VA	0.38 ± 0,35 logMAR	0.35 ± 0,29 logMAR
					Number of recurrences	4 (12.5%)	5 (14.7%)
					Improvement in VA (gaining > = 2 Snellen lines)	27/32	28/34
					Ocular inflammation (grade 0/trace)	28 (87.5%)	28 (82.4%)
					Retinal lesion size improvement	21 (65.6%)	23 (67.6%)
					Adverse drug reactions (major)	0	1 (2.9%)
**Bosch-Driessen, 2002**	Netherlands	*N* = 46 Intervention = 24 Control = 22	PYR + Azithromycin + folinic acid + prednisone Administration route: oral	PYR/SDZ + folinic acid + prednisone Administration route: oral	Changes in VA	NR	NR
					Number of recurrences (at least one year follow up)	5 /15 (33%)	5 / 9 (56%)
					Improvement in VA (> = 0.5 logMAR units at 3 months)	5 /24 (21%)	5/18 (28%)
					Ocular inflammation (No inflammatory cells from vitreous within 4 w)	14 /20 (70%)	10/14 (71%)
					Retinal lesion size improvement (decrease more than 0,5 optic disk diameter in 3 months)	9/ 22 (41%)	7 /17 (41%)
					Adverse drug reactions (all)	8/ 24 (33%)	14 /22 (64%)
**Colin, 1989**	Not exactly reported	*N* = 29 Intervention = 14 Control = 15	Clindamycin Administration route: subconjunctival injections + oral prednisolone	PYR/SDZ + Prednisolone Administration route: oral Folinic acid Administration route: Intramuscular	Changes in VA Ocular inflammation Improvement in VA Retinal lesion Size	NR NR NR NR	NR NR NR NR
					Number of recurrences	21%	36%
					Adverse drug reactions (all)	1	1
**Ghavidel, 2017**	Iran	*N* = 72 Intervention = 36 Control = 36	Azithromycin + prednisolone Administration route: oral	PYR/SDZ + prednisolone Administration route: oral	Changes in VA Ocular inflammation	0.35 logMAR (20/44 Snellen acuity) NR	0.39 logMAR (20/49 Snellen acuity) NR
					Improvement in VA	NR	NR
					Number of recurrences (during 24 months after treatment)	18 (50%)	4 (11,1%)
					Retinal lesion size (reduction during treatment)	354.86 µm	638.89 µm
					Adverse drug reactions (all)	4 (12.5%)	20 (55.5%)
**Kartasasmita, 2017**	Indonesia	*N* = 28 Intervention = 14 Control = 14	TMP/SMX + Clindamycin + methylprednisolone Administration route: oral	PYR/SDZ + methylprednisolone + folic acid Administration route: oral	Changes in VA Number of recurrences Improvement in VA Ocular inflammation Adverse drug reactions	NR NR NR NR NR	NR NR NR NR NR
					Retinal lesion size (percentage of lesion area reduction in the third w)	57.50%	52.5%
**Lashay, 2017**	Iran	*N* = 27 Intervention = 14 Control = 13	Azithromycin + prednisone Administration route: oral	TMP/SMX + Prednisone Administration route: oral	Changes in VA	0.24 ± 0.04 logMAR	0.30 ± 0.01 logMAR
					Number of recurrences Improvement in VA	NR 10/14	NR 10/13
					Ocular inflammation (vitreous inflammatory cells clearance)	7 (50%)	10 (77%)
					Retinal lesion size (reduction)	24.2% ± 6,5%	36.6% ± 4,6%
					Adverse drug reactions (mild)	4 (28.5%)	3 (23%)
**Ortega, 2000**	Mexico	*N* = 46 Intervention G1 = 13 Intervention G2 = 22 Intervention G3 = 11	G1 TMP/SMX + PYR + prednisone + folinic acid G2 TMP/SMX + Clindamycin + prednisone Administration route: oral G3 TMP/SMX + PYR + Clindamycin + prednisone + folinic acid Administration route: oral	-	Changes in VA Improvement in VA Ocular inflammation Retinal lesion size	NR NR NR NR	- - - -
					Number of relapses	G1 1 (7.6%) G2 5 (31.2%) G3 4 (36.3%)	
					Adverse drug reactions (called “Complications from treatment” and apparently mild)	G1 1 (7.6%) G2 4 (18.1%) G3 0 (0%)	-
**Soheilian, 2005**	Iran	*N* = 59 Intervention = 30 Control = 29	TMP/SMX + oral prednisolone Administration route: oral	PYR/SDZ + folinic acid + prednisolone Administration route: oral	Improvement in VA	NR	NR
					Changes in VA (increase)	0,52 logMAR	0,56 logMAR
					Number of recurrences	3 (10%)	3 (10,3%)
					Ocular inflammation (Reduction of vitreous inflammatory cells (0–trace cells) 6 w after treatment)	17 (56,7%)	20 (69%)
					Retinal lesion size (mean reduction 6 w after treatment)	59%	61%
					Adverse drug reactions	1^a^/31 (2,8%)	1^a^/30 (2,9%)
**Soheilian, 2011**	Iran	*N* = 68 Intervention = 34 Control = 34	Clindamycin + dexamethasone Administration route: intravitreal	PYR + SDZ + folinic acid + prednisolone Administration route: oral	Improvement in VA	NR	NR
					Changes in VA (increase)	0,44 ± 0,24 logMAR	0,29 ± 0,19 logMAR
					Number of recurrences (eyes)	2	2
					Ocular inflammation (trace or no vitreous cells)	15 (51,7%)	15 (55,5%)
					Retinal lesion size (calculated in pixels using MATLAB environment)	116,994 +—143,997 Pixels	89,606 +—651,553 pixels
					Adverse drug reactions (all)	4/34	2^a^/36

*PYR* Pyrimethamine, *SDZ* Sulfadiazine, *TMP/SMX* trimethoprim/ sulfamethoxazole, *N* Total sample, *NR* Not reported, *VA* Visual Acuity, w: weeks. ^a^ Patients excluded from the study. See details in Annex 3

### Risk of bias

**Fig. 2:**
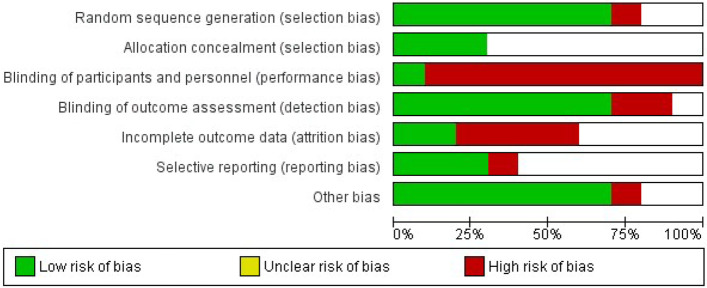
Risk of bias graph: review authors’ judgments regarding each risk of bias item, presented as percentages across all included studies

**Fig. 3:**
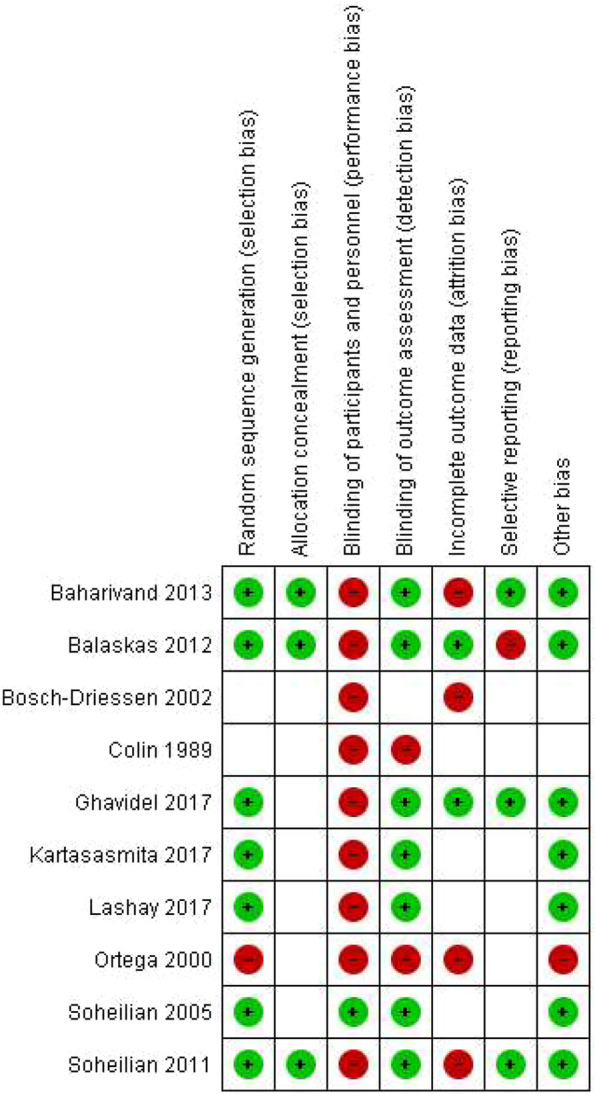
Risk of bias summary: review authors’ judgments regarding each risk of bias item for each included study

Overall, nine studies had a high risk of performance bias; seven studies had low risks of selection bias (random sequence generation), detection bias, and other bias. The Ortega 2000 [43] study had a high risk of bias in most attributes; the Soheilian 2005 [30] study did not have a high risk of bias in any evaluated attributes. The bias risk assessments for all included studies are summarized in Figs. 2 and 3.

It was not possible to assess the publication bias since the number of studies necessary to evaluate it was not enough.

### Effects of interventions

#### Intravitreal clindamycin versus PYR/SDZ

**Fig. 4:**
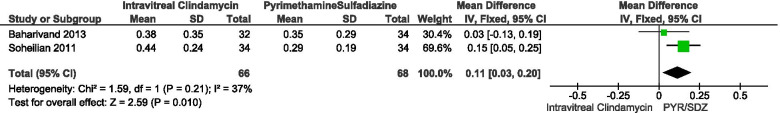
Comparison between intravitreal clindamycin and PYR/SDZ, mean changes in visual acuity

**Fig. 5:**
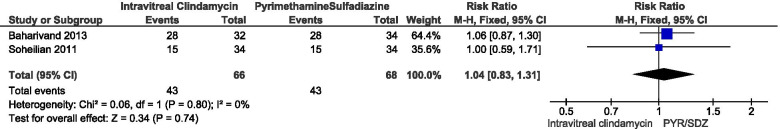
Comparison between intravitreal clindamycin and PYR/SDZ, resolution of vitreous inflammation

The number of studies that provided data for this comparison was two RCT, and both were included in the meta-analysis. The Soheilian 2011 [44] and Baharivand 2013 [37] studies compared head-to-head intravitreal clindamycin versus PYR/SDZ. Regarding changes in visual acuity, the meta-analysis revealed statistically significant differences in favor of intravitreal clindamycin (mean difference = 0.11 logMAR; 95% CI = 0.03 to 0.20) (Fig. 4). However, this result is not considered clinically relevant, as will be discussed below. Additionally, in the random-effects model, there were no statistically significant differences (MD 0.10 logMAR; 95% CI = -0.01 to 0.22). No significant differences were reported in the two studies regarding the number of recurrences and lesion size. In the Soheilian 2011 [44] study, lesion size was reported in pixels with a mean percentage of changes of 1.4% (95% CI = -14.6% to 17.4%, *p =* 0.86). In contrast, in the Baharivand 2013 [37] study, retinal lesion size was reported as ≤ 1 disc area (DA), < 1 and ≤ 2 DA, and < 2 and ≤ 3 DA. Baharivand et al. [37] regarded improvement in retinal lesion size as a lesion size that decreased in terms of DA classification (improvement of 65.6% in intravitreal clindamycin group versus 67.6% in PYR/SDZ group, *p =* 0.86). Although none of the studies reported ocular inflammation according to the SUN, this outcome was evaluated as a reduction of vitreous cells to the level of 0 or trace after treatment, which was regarded as the resolution of vitreous inflammation. Thus, for this outcome, there was no statistical heterogeneity (I^2^ = 0%), and no significant differences were found between the treatment groups (RR = 1.04; 95% CI = 0.83 to 1.31) (Fig. 5). In the Baharivand 2013 study, the arm that evaluated intravitreal clindamycin presented no adverse drug reactions, whereas in the PYR/SDZ arm, one case of adverse drug reactions was reported (hepatotoxicity). In the Soheilian 2011 study, there were two cases of adverse drug reactions in the PYR/SDZ arm (skin rash and thrombocytopenia), which were not included in the efficacy analysis, while in the intravitreal clindamycin arm, there were four cases of adverse drug reactions (three subconjunctival hemorrhages and one transient raised intraocular pressure).

#### TMP/SMX versus another antibiotic

**Fig. 6:**
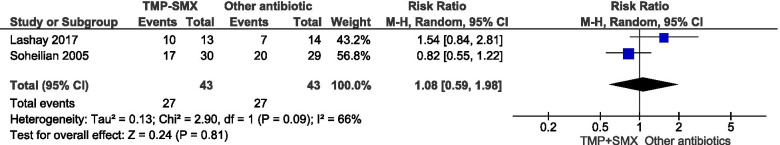
Comparison between TMP/SMX and another antibiotic, resolution of vitreous inflammation

Two RCTs compared TMP/SMX versus another antibiotic, and both were included in the meta-analysis. The Soheilian 2005 and Lashay 2017 [42] studies compared TMP/SMX versus PYR/SDZ and TMP/SMX versus azithromycin, respectively. In terms of ocular inflammation, regarded as the percentage of patients with a reduction of vitreous inflammatory cells (0 or trace cells) after treatment, there were no significant differences (RR = 1.08; 95% CI = 0.59 to 1.98) with moderate heterogeneity (I^2^ = 66%) (Fig. 6). Likewise, both treatments showed a similar number of adverse drug reactions that ranged from mild to significant. A summary of the results can be found in Table 1. Annex 3 provides detailed information.

#### Other interventions

Six out of ten studies included data about other therapeutic schemes, and none were included in the meta-analysis.

The Kartasasmita 2017 [41] study compared TMP/SMX + oral clindamycin versus PYR/SDZ for 3 weeks and found that the mean percentage of lesion remission from the first visit to the last visit was 57.5% in the first group, while it was 52.5% in the second group (*p* = 0.72). This result was obtained by comparing the reduction in retinal lesion size between the pretreatment evaluation and the third week of treatment evaluation. However, in the first week of treatment, the authors found that the mean percentage of patients with lesion remission was higher in the TMP/SMX + oral clindamycin group than in the PYR/SDZ group (71.7% versus 19.8%; *p* = 0.001). There were no safety considerations in this study.

The Ghavidel 2017 [40] study compared azithromycin versus PYR/SDZ for 6 weeks. The authors reported that visual acuity improved by 0.35 logMAR in the azithromycin group and 0.39 logMAR in the PYR/SDZ group, although this difference was not statistically significant (*p* = 0.33). In addition, there was no difference in the size of the retinal lesion detected. In terms of safety, only four patients in the azithromycin group had gastrointestinal adverse drug reactions. For the PYR/SDZ group, 20 patients presented adverse drug reactions (16 gastrointestinal and 4 dizziness) (*p* < 0.01 compared with the azithromycin group); however, no severe adverse drug reactions were observed, such as bone marrow suppression.

The Bosch-Driessen 2002 [38] study compared pyrimethamine + azithromycin (PYR/AZ) versus PYR/SDZ for 4 weeks. This study did not report explicit baseline data for included patients. There were no significant differences in visual acuity improvement at 3 months, as well as in the number of recurrences to the first year of treatment or the disappearance of inflammatory cells from vitreous within 4 weeks. The frequency of adverse drug reactions was higher in the PYR/SDZ group than in the PYR/AZ group (64% versus 33%, respectively; *p* = 0.04); more patients discontinued treatment in the PYR/SDZ group (14%), compared with the PYR/AZ group (0%) (*p* = 0.1).

The Balaskas 2012 [36] study compared azithromycin with PYR/SDZ in 19 patients without a specific duration of treatment, with a follow-up of 3 months. This study investigated changes in lesion size, time of the sharpening of lesion borders, time to scarring, and time to disease inactivity in days; these results did not demonstrate statistically significant differences. Skin rash was observed in one patient in the PYR/SDZ group. Treatment failure was documented in one patient in the azithromycin group.

The Colin 1989 [39] study compared subconjunctival injections of clindamycin (3 weeks of treatment) versus PYR/SDZ (6 weeks of treatment) in 29 patients. The authors reported visual acuity but did not specify the measurement units or whether they represented near or far visual acuity. At 14 months after treatment, there were 21% recurrences in the clindamycin group and 36% in the PYR/SDZ group (*p*-value not reported). In the PYR/SDZ group, one patient exhibited Stevens-Johnson syndrome, and another patient exhibited a skin rash. In the clindamycin group, one patient exhibited corneal ulceration with inflammation.

The Ortega 2000 [43] study was an RCT with three treatment groups: TMP/SMX + PYR versus TMP/SMX + clindamycin versus TMP/SMX + PYR + clindamycin for 8 weeks. All groups exhibited an increase in visual acuity (*p* < 0.05) compared with baseline. Notably, only the TMP/SMX + clindamycin group required less time to resolve inflammation (*p* = 0.044) and achieve healing (*p* = 0.016). Although there were adverse drug reactions, these were not reported explicitly. Table 1 synthesized the results of the included studies in this systematic review.

## Discussion

### Agreements and disagreements with other studies or reviews

Our meta-analysis showed that, when comparing the effectiveness of intravitreal clindamycin and oral PYR/SDZ, there were significant differences in visual acuity in favor of intravitreal clindamycin (Fig. 4). However, there were no clinically significant differences in the use of either treatment because the changes in visual acuity corresponded to 0.1 logMAR (i.e., one line in the Snellen chart). Significant changes in visual acuity are defined as worsening or gaining  ≥ 15 letters (i.e., three lines in standardized optotypes) [45, 46]. It is important to highlight the findings of the meta-analysis by Pradhan et al. in 2016 [13], where only one study (Felix et al. 2014) [47] evaluated visual acuity outcome measured at least 3 months after the start of treatment, showing similar changes in the TMP/SMX and placebo groups [13].

Regarding lesion size, the measurement methods in the included studies did not allow a meta-analytical comparison of the findings. Furthermore, in the meta-analysis by Pradhan et al., the outcome of lesion size was considered, but the analyzed studies did not report it [13]. The methods described in the literature for measuring lesion size include the following: fundoscopic images, in which the lesion and optic disc areas were measured using Photoshop software and the ratio of the lesion to optic disc area was calculated [48]; a program written in the MATLAB environment made to determine the margin of the retinitis and to measure the area of interest in pixels [44]; and the retinal lesion size in terms of ≤ 1 disc area (DA), < 1 and ≤ 2 DA, and < 2 and ≤ 3 DA. “Improvement” was considered a lesion size that decreased in terms of DA classification [37]. It is important to create consensus regarding lesion size measurements and the percentage of lesion reduction in future investigations.

Even though the SUN working group was first published in 2005 [49], intraocular inflammation measures were not used in most of the included studies, and this outcome could not be objectively measured. Therefore, in further studies, the use of this classification is crucial to standardize and compare the intraocular inflammation outcomes.

Concerning the antibiotic interventions evaluating recurrences in OT, it is important to notice that all the regimens of antibiotics were not included in our systematic review. For example, an observational study performed in patients treated with intensive therapy in the active phase, followed by long-term treatment with pyrimethamine sulfadoxine (PYR/SDX) (it was not included in our review since it was not a RCT), revealed a 90.9% probability of 3-year recurrence-free survival after the first intervention [50]. Future RCTs may include PYR/SDX scheme, as it is one of the alternative therapies accessible in different countries where PYR/SDZ is not available.

Regarding the number of recurrences, there were no statistically significant differences between PYR/SDZ and intravitreal clindamycin in the evaluated studies. Nevertheless, the reduction of recurrences in patients with OT has been evaluated in long-term therapy studies after the acute stage of infection has been controlled. In the meta-analysis by Pradhan et al., the authors found that treatment with antibiotics compared with placebo or no treatment probably reduces the risk of recurrent toxoplasma retinochoroiditis (Risk ratio 0.26, 95% CI 0.11 to 0.63; 227 participants; 3 studies; I^2^ = 0%; moderate-quality evidence).

Additionally, in the meta-analysis by Pradhan et al., there was low-quality evidence regarding visual acuity changes, intraocular inflammation, and adverse drug reactions [13]. This agrees with the results of our metanalysis, where we found that the evidence quality of the included studies was from low to very low (GRADE methodology results are shown in Annex 2). This means that there are no well-conducted RCTs regarding OT treatment, indicating that further rigorous research is very likely to have an important impact on the confidence of the estimated effect and, in consequence, could change it.

Zhang et al. [19] published a network meta-analysis about treatment in ocular toxoplasmosis. The overall confidence rating of Zhang et al. review is critically low, according to the AMSTAR 2 (A Measurement Tool to Assess Systematic Reviews 2) [51]. Indeed, more than one critical flaw was found in the AMSTAR 2 assessment in domains 2, 7, 9, 13, and 15. Notably, the network meta-analysis of Zhang et al. did not report the risk of bias of the included studies. In contrast, our study accomplishes all the items required in AMSTAR 2, obtaining high overall confidence. Furthermore, our systematic review and meta-analysis provide an accurate and comprehensive summary of the results of the available studies that address the question of interest. Nonetheless, we recognize that although the AMSTAR 2 was not intended to handle the special requirements of a network meta-analysis [51], a modified AMSTAR should be developed to assess network meta-analyses [52]. Recently published papers showed that the tool called CINeMA (Confidence in Network Meta-Analysis) contributes to assessing the credibility of the results of a network meta-analysis and should be taken into account when properly evaluating this type of study design [53, 54]. A comparison of AMSTAR 2 results of our work and Zhang et al.'s study is available in Annex 4.

Regarding the safety of the analyzed drugs, it was found that those studies that reported information on PYR/SDZ adverse drug reaction had a frequency that was in the range of 0.05% (Sohelian 2011 [44]) to 100% (Balaskas 2012 [36]), including all degrees of severity. In the same way, concerning TMP/SMX, adverse drug reaction was reported from 2.8% (Sohelian 2005 [30]) to 23% (Lashay 2017 [42]), and through various degrees of severity. Thus, overall, there were no significant differences in adverse drug reactions for the different treatment regimens compared to each other, except for the azithromycin-containing arms. Indeed, Ghavidel et al. [40] found a better tolerance in the azithromycin group than its comparator, which also occurred in Bosch-Driessen 2002 [38]. Therefore, azithromycin appears to have a better safety profile among the different options analyzed through the studies included in this systematic review.

Finally, according to the results of this systematic review, some combinations of antibiotic schemes have not been compared in clinical trials but are used in the clinical practice. Therefore, we consider that it is crucial to know the results of comparisons between combined clindamycin + TMP / SMX + prednisone versus TMP / SMX + prednisone versus clindamycin + prednisone. Also, quadruple-drug therapies such as TMP / SMX + clindamycin + prednisone versus PYR / SDZ + azithromycin + prednisone. We also consider it relevant to compare sulfadiazine and sulfadoxine in well-conducted clinical trials, to understand the superiority or noninferiority between these sulfa drugs in OT.

### Potential biases in the review process and limitations

We aimed to minimize potential biases by conducting a highly sensitive search for trials; we followed rigorous methods as recommended by the Cochrane group “Risk of Bias” tool for RCTs and other criteria considered in the Cochrane Handbook for Systematic Reviews of Interventions [29].

Most primary outcomes established in the study protocol were not settled in the articles found by the search strategy; thus, secondary outcomes were considered for the quantitative analysis [21]. In addition, missing data and supplementary material were requested from some authors; however, they did not respond or did not have access to those data.

## Conclusions

In general, the quality of the evidence found was low to very low. Although visual acuity analysis revealed a statistically significant difference in the first comparison of intravitreal clindamycin and oral PYR/SDZ, the result was not clinically relevant. Regarding the remaining outcomes, none of them showed statistically significant differences (i.e., no treatments were superior to others). Therefore, the selection of treatment regimen must be performed on an individual basis, considering the safety of each therapeutic regimen, (azithromycin appears to have the best safety profile), medical history of sulfa allergy, and the availability of medications offered within each nation’s health system. Although there is no consensus regarding the best treatment regimens in OT, the treatment should include at least two antibiotics along with corticosteroids.

## Supplementary Information

**Additional file 1.**

**Additional file 2.**

**Additional file 3.**

**Additional file 4.**

## Data Availability

The data that support the findings of this study are available from the corresponding author, ADLT, upon reasonable request.

## Abbreviations

OT: Ocular toxoplasmosis
PYR/SDZ: Pyrimethamine + sulfadiazine
PYR/SDX: Pyrimethamine + sulfadoxine
PYR/AZ: Pyrimethamine + azithromycin
TMP/SMX: Trimethoprim + sulfamethoxazole
SUN: Standardization of Uveitis Nomenclature
