# Supplementary material for: Safety and efficacy of different antibiotic regimens in patients with ocular toxoplasmosis: systematic review and meta-analysis
Source: Syst Rev. 2021 Jul 19;10:206. doi: 10.1186/s13643-021-01758-7 (PMC8287816; doi:10.1186/s13643-021-01758-7)
Supplement: Supplementary file 1 — Additional file 1. [file 13643_2021_1758_MOESM1_ESM.docx]

**ANNEX 1**

**FIRST SEARCH STRATEGY (MARCH 2018)**

| **Search electronic report #1** | |
| --- | --- |
| **Search type** | New |
| **Databases** | - Ovid MEDLINE(R) <1946 to March Week 3 2018>, Ovid MEDLINE(R) In-Process & Other Non-Indexed Citations <March 26, 2018>, Ovid MEDLINE(R) Daily Update <March 26, 2018> |
| **Platform** | Ovid.com |
| **Search date** | 27/03/2018 |
| **Update date** | Undefined |
| **Range of search date** | None |
| **Language restrictions** | None |
| **Other limits** | RCT |
| **Search strategy (results)** | 1 exp Toxoplasmosis, Ocular/ (1726)  2 exp Toxoplasma/ (12395)  3 exp Toxoplasmosis/ (18840)  4 toxoplasm$.tw. (25351)  5 gondi$.tw. (13648)  6 exp Chorioretinitis/ (2334)  7 exp Retinitis/ (7400)  8 exp Choroiditis/ (3548)  9 exp Uveitis/ (28499)  10 retinochoroiditis.tw. (569)  11 chorioretin$.tw. (5735)  12 uveitis.tw. (15622)  13 ocular.tw. (109171)  14 retinitis.tw. (10694)  15 choroidoretinitis.tw. (19)  16 choroiditis.tw. (1080)  17 eye.tw. (211888)  18 ophthalmic.tw. (27839)  19 2 or 3 or 4 or 5 (27834)  20 6 or 7 or 8 or 9 or 10 or 11 or 12 or 13 or 14 or 15 or 16 or 17 or 18 (335678)  21 19 and 20 (2724)  22 1 or 21 (3035)  23 exp Anti-Infective Agents/ (1483719)  24 exp Trimethoprim, Sulfamethoxazole Combination/ (6450)  25 tmp-smx.tw. (1046)  26 (tmp adj5 smx).tw. (1168)  27 cotrimoxazole.tw. (2884)  28 trimethoprim$.tw. (15020)  29 exp Pyrimethamine/ (4505)  30 pyrimethamine.tw. (4599)  31 exp Sulfadoxine/ (2078)  32 sulfadoxine.tw. (1899)  33 exp Sulfadiazine/ (3565)  34 sulfadiazine.tw. (2780)  35 exp Clindamycin/ (5378)  36 clindamycin.tw. (9349)  37 exp Tetracyclines/ (45334)  38 tetracycline$.tw. (32587)  39 exp Minocycline/ (5392)  40 minocycline.tw. (5868)  41 exp Clarithromycin/ (5770)  42 clarithromycin.tw. (8090)  43 exp Azithromycin/ (4465)  44 azithromycin.tw. (6769)  45 exp Atovaquone/ (688)  46 atovaquone.tw. (881)  47 exp Spiramycin/ (683)  48 spiramycin.tw. (1213)  49 exp Rifabutin/ (2049)  50 rifabutin.tw. (1089)  51 exp Trimetrexate/ (361)  52 trimetrexate.tw. (446)  53 exp Lincomycin/ (7114)  54 lincomycin.tw. (2341)  55 exp Dapsone/ (4634)  56 dapsone.tw. (3937)  57 exp Sulfisoxazole/ (942)  58 sulfafurazole.tw. (31)  59 sulfisoxazole.tw. (643)  60 exp Ciprofloxacin/ (12115)  61 ciprofloxacin.tw. (22336)  62 exp Doxycycline/ (8777)  63 doxycycline.tw. (11581)  64 exp Miocamycin/ (278)  65 mio?amycin.tw. (140)  66 exp Erythromycin/ (23562)  67 erythromycin.tw. (19385)  68 exp Macrolides/ (100719)  69 macrolide$.tw. (13983)  70 exp Sulfonamides/ (112276)  71 sulfonamide$.tw. (13056)  72 exp Sulfamerazine/ (452)  73 sulfamerazine.tw. (351)  74 exp Nifurtimox/ (414)  75 nifurtimox.tw. (647)  76 exp Methotrexate/ (35401)  77 methotrexate.tw. (36525)  78 23 or 24 or 25 or 26 or 27 or 28 or 29 or 30 or 31 or 32 or 33 or 34 or 35 or 36 or 37 or 38 or 39 or 40 or 41 or 42 or 43 or 44 or 45 or 46 or 47 or 48 or 49 or 50 or 51 or 52 or 53 or 54 or 55 or 56 or 57 or 58 or 59 or 60 or 61 or 62 or 63 or 64 or 65 or 66 or 67 or 68 or 69 or 70 or 71 or 72 or 73 or 74 or 75 or 76 or 77 (1640681)  79 randomized controlled trial.pt. (456158)  80 controlled clinical trial.pt. (92251)  81 random$.ab. (917871)  82 placebo.ab. (184482)  83 clinical trials as topic.sh. (183012)  84 randomly.ab. (281816)  85 trial.ti. (175563)  86 79 or 80 or 81 or 82 or 83 or 84 or 85 (1339613)  87 exp animals/ not humans.sh. (4436130)  88 86 not 87 (1223836)  89 22 and 78 and 88 (43) |
| **# of records identified** | 43 |

| **Search electronic report #2** | |
| --- | --- |
| **Search type** | New |
| **Databases** | - Embase |
| **Platform** | Embase.com |
| **Search date** | 28/03/2018 |
| **Update date** | Undefined |
| **Range of search date** | None |
| **Language restrictions** | None |
| **Other limits** | RCT |
| **Search strategy (results)** | #1.'oculartoxoplasmosis'/exp145  #2.'toxoplasma'/exp17,656  #3.'toxoplasmosis'/exp23,426  #4.toxoplasm*:ab,ti29,363  #5.gondi*:ab,ti15,352  #6.'chorioretinitis'/exp3,545  #7.'retinitis'/exp27,116  #8.'choroiditis'/exp6,838  #9.'uveitis'/exp51,962  #10.retinochoroiditis:ab,ti661  #11.chorioretin*:ab,ti7,037  #12.uveitis:ab,ti20,932  #13.ocular:ab,ti135,644  #14.retinitis:ab,ti12,903  #15.choroidoretinitis:ab,ti19  #16.choroiditis:ab,ti1,229  #17.eye:ab,ti262,343  #18.ophthalmic:ab,ti37,191  #19.#2OR#3OR#4OR#536,038  #20.#6OR#7OR#8OR#9OR#10OR#11OR#12OR#13427,857  OR#14OR#15OR#16OR#17OR#18  #21.#19AND#203,762  #22.#1OR#213,778  #23.'antiinfectiveagent'/exp2,903,092  #24.'cotrimoxazole'/exp72,129  #25.'tmpsmx':ab,ti1,553  #26.(tmpNEAR/5smx):ab,ti1,748  #27.cotrimoxazole:ab,ti4,112  #28.trimethoprim*:ab,ti18,899  #29.'pyrimethamine'/exp9,892  #30.pyrimethamine:ab,ti5,447  #31.'sulfadoxine'/exp3,503  #32.sulfadoxine:ab,ti2,312  #33.'sulfadiazine'/exp8,282  #34.sulfadiazine:ab,ti3,335  #35.'clindamycin'/exp45,935  #36.clindamycin:ab,ti12,226  #37.'tetracyclinederivative'/exp157,574  #38.tetracycline*:ab,ti38,028  #39.'minocycline'/exp21,615  #40.minocycline:ab,ti7,979  #41.'clarithromycin'/exp33,240  #42.clarithromycin:ab,ti11,948  #43.'azithromycin'/exp29,739  #44.azithromycin:ab,ti10,282  #45.'atovaquone'/exp3,128  #46.atovaquone:ab,ti1,184  #47.'spiramycin'/exp4,271  #48.spiramycin:ab,ti1,375  #49.'rifabutin'/exp5,529  #50.rifabutin:ab,ti1,418  #51.'trimetrexate'/exp1,261  #52.trimetrexate:ab,ti492  #53.'lincomycin'/exp8,348  #54.lincomycin:ab,ti2,520  #55.'dapsone'/exp17,600  #56.dapsone:ab,ti5,491  #57.'sulfafurazole'/exp3,797  #58.sulfafurazole:ab,ti47  #59.sulfisoxazole:ab,ti726  #60.'ciprofloxacin'/exp86,495  #61.ciprofloxacin:ab,ti30,482  #62.'doxycycline'/exp46,027  #63.doxycycline:ab,ti16,398  #64.'miokamycin'/exp450  #65.mio?amycin:ab,ti174  #66.'erythromycin'/exp70,510  #67.erythromycin:ab,ti23,312  #68.'macrolide'/exp201,974  #69.macrolide*:ab,ti18,596  #70.'sulfonamide'/exp167,394  #71.sulfonamide*:ab,ti14,487  #72.'sulfamerazine'/exp1,372  #73.sulfamerazine:ab,ti384  #74.'nifurtimox'/exp1,743  #75.nifurtimox:ab,ti769  #76.'methotrexate'/exp161,008  #77.methotrexate:ab,ti57,646  #78.#23OR#24OR#25OR#26OR#27OR#28OR#29OR3,009,273  #30OR#31OR#32OR#33OR#34OR#35OR#36OR  #37OR#38OR#39OR#40OR#41OR#42OR#43OR  #44OR#45OR#46OR#47OR#48OR#49OR#50OR  #51OR#52OR#53OR#54OR#55OR#56OR#57OR  #58OR#59OR#60OR#61OR#62OR#63OR#64OR  #65OR#66OR#67OR#68OR#69OR#70OR#71OR  #72OR#73OR#74OR#75OR#76OR#77  #79.('randomizedcontrolledtrial'/expOR'single1,478,255  blindprocedure'/expOR'doubleblind  procedure'/expOR'crossoverprocedure'/expOR  random*:ab,tiORplacebo*:ab,tiORallocat*:ab,ti  ORcrossover*:ab,tiOR'crossover':ab,tiOR  trial:tiOR((doubl*NEXT/1blind*):ab,ti))NOT  (('animal'/deOR'animalexperiment'/deOR  'nonhuman'/de)NOT(('animal'/deOR'animal  experiment'/deOR'nonhuman'/de)AND'human'/de))  #80.#22AND#78AND#7950  #81.#22AND#78AND#79AND[embase]/lim44 |
| **# of records identified** | 44 |

| **Search electronic report #3** | |
| --- | --- |
| **Search type** | New |
| **Databases** | - EBM Reviews - Cochrane Central Register of Controlled Trials <February 2018> |
| **Platform** | Ovid.com |
| **Search date** | 27/03/2018 |
| **Update date** | Undefined |
| **Range of search date** | None |
| **Language restrictions** | None |
| **Other limits** | None |
| **Search strategy (results)** | 1 exp Toxoplasmosis, Ocular/ (14)  2 exp Toxoplasma/ (19)  3 exp Toxoplasmosis/ (72)  4 toxoplasm$.tw. (177)  5 gondi$.tw. (54)  6 exp Chorioretinitis/ (16)  7 exp Retinitis/ (110)  8 exp Choroiditis/ (25)  9 exp Uveitis/ (429)  10 retinochoroiditis.tw. (21)  11 chorioretin$.tw. (185)  12 uveitis.tw. (591)  13 ocular.tw. (8052)  14 retinitis.tw. (285)  15 choroidoretinitis.tw. (0)  16 choroiditis.tw. (6)  17 eye.tw. (14701)  18 ophthalmic.tw. (2816)  19 2 or 3 or 4 or 5 (179)  20 6 or 7 or 8 or 9 or 10 or 11 or 12 or 13 or 14 or 15 or 16 or 17 or 18 (21339)  21 19 and 20 (45)  22 1 or 21 (46)  23 exp Anti-Infective Agents/ (51513)  24 exp Trimethoprim, Sulfamethoxazole Combination/ (1)  25 tmp-smx.tw. (169)  26 (tmp adj5 smx).tw. (179)  27 cotrimoxazole.tw. (326)  28 trimethoprim$.tw. (1182)  29 exp Pyrimethamine/ (557)  30 pyrimethamine.tw. (898)  31 exp Sulfadoxine/ (446)  32 sulfadoxine.tw. (564)  33 exp Sulfadiazine/ (222)  34 sulfadiazine.tw. (232)  35 exp Clindamycin/ (691)  36 clindamycin.tw. (1166)  37 exp Tetracyclines/ (1901)  38 tetracycline$.tw. (1438)  39 exp Minocycline/ (309)  40 minocycline.tw. (573)  41 exp Clarithromycin/ (1136)  42 clarithromycin.tw. (2116)  43 exp Azithromycin/ (758)  44 azithromycin.tw. (1505)  45 exp Atovaquone/ (68)  46 atovaquone.tw. (105)  47 exp Spiramycin/ (26)  48 spiramycin.tw. (76)  49 exp Rifabutin/ (74)  50 rifabutin.tw. (119)  51 exp Trimetrexate/ (12)  52 trimetrexate.tw. (20)  53 exp Lincomycin/ (731)  54 lincomycin.tw. (61)  55 exp Dapsone/ (212)  56 dapsone.tw. (318)  57 exp Sulfisoxazole/ (67)  58 sulfafurazole.tw. (7)  59 sulfisoxazole.tw. (53)  60 exp Ciprofloxacin/ (966)  61 ciprofloxacin.tw. (1765)  62 exp Doxycycline/ (768)  63 doxycycline.tw. (1240)  64 exp Miocamycin/ (17)  65 mio?amycin.tw. (14)  66 exp Erythromycin/ (2730)  67 erythromycin.tw. (1461)  68 exp Macrolides/ (6231)  69 macrolide$.tw. (680)  70 exp Sulfonamides/ (8908)  71 sulfonamide$.tw. (111)  72 exp Sulfamerazine/ (5)  73 sulfamerazine.tw. (1)  74 exp Nifurtimox/ (6)  75 nifurtimox.tw. (16)  76 exp Methotrexate/ (3071)  77 methotrexate.tw. (5840)  78 23 or 24 or 25 or 26 or 27 or 28 or 29 or 30 or 31 or 32 or 33 or 34 or 35 or 36 or 37 or 38 or 39 or 40 or 41 or 42 or 43 or 44 or 45 or 46 or 47 or 48 or 49 or 50 or 51 or 52 or 53 or 54 or 55 or 56 or 57 or 58 or 59 or 60 or 61 or 62 or 63 or 64 or 65 or 66 or 67 or 68 or 69 or 70 or 71 or 72 or 73 or 74 or 75 or 76 or 77 (70426)  79 22 and 78 (32) |
| **# of records identified** | 32 |

| **Search electronic report #4** | |
| --- | --- |
| **Search type** | New |
| **Databases** | - Lilacs Via iAHx |
| **Platform** | Lilacs.bvsalud.org |
| **Search date** | 27/03/2018 |
| **Update date** | Undefined |
| **Range of search date** | None |
| **Language restrictions** | None |
| **Other limits** | Clinical Trials |
| **Search strategy (results)** | (mh:("Toxoplasmosis, Ocular")) OR (ti:(Toxoplasmosis)) OR (ab:(Toxoplasmosis)) AND db:("LILACS") AND type_of_study:("clinical_trials") |
| **# of records identified** | 12 |

- **Total of records identified**

**MEDLINE + EMBASE + COCHRANE CENTRAL + LILACS = 43+44+32+12 = 131**

- **Number of records without duplicates = 94**

|  | **Other sources: electronic report #5** | | |
| --- | --- | --- | --- |
| **Database / Date** | **Date** | **Search Strategy** | **Results** |
| clinicaltrials.gov | March 2018 | ocular toxoplasmosis | 4 |
| WHO International Clinical Trials Registry. | March 2018 | ocular toxoplasmosis | 6 |
| OPEN GREY | March 2018 | ocular toxoplasmosis | 8 |

**SEARCH STRATEGY UPDATE (NOVEMBRE 2020)**

| **Search electronic report #1** | |
| --- | --- |
| **Search type** | UPDATE |
| **Databases** | - Ovid MEDLINE(R) ALL <1946 to November 20, 2020> |
| **Platform** | Ovid.com |
| **Search date** | 21/11/2020 |
| **Update date** | Undefined |
| **Range of search date** | 2018-2020 |
| **Language restrictions** | None |
| **Other limits** | RCT |
| **Search strategy (results)** | 1 exp Toxoplasmosis, Ocular/ (1837)  2 exp Toxoplasma/ (13869)  3 exp Toxoplasmosis/ (20247)  4 toxoplasm$.tw. (28365)  5 gondi$.tw. (15978)  6 exp Chorioretinitis/ (2460)  7 exp Retinitis/ (7784)  8 exp Choroiditis/ (3770)  9 exp Uveitis/ (31040)  10 retinochoroiditis.tw. (648)  11 chorioretin$.tw. (6966)  12 uveitis.tw. (18321)  13 ocular.tw. (127925)  14 retinitis.tw. (12410)  15 choroidoretinitis.tw. (20)  16 choroiditis.tw. (1266)  17 eye.tw. (249873)  18 ophthalmic.tw. (33872)  19 2 or 3 or 4 or 5 (30854)  20 6 or 7 or 8 or 9 or 10 or 11 or 12 or 13 or 14 or 15 or 16 or 17 or 18 (392145)  21 19 and 20 (3048)  22 1 or 21 (3367)  23 exp Anti-Infective Agents/ (1643240)  24 exp Trimethoprim, Sulfamethoxazole Combination/ (6965)  25 tmp-smx.tw. (1222)  26 (tmp adj5 smx).tw. (1378)  27 cotrimoxazole.tw. (3231)  28 trimethoprim$.tw. (17317)  29 exp Pyrimethamine/ (4708)  30 pyrimethamine.tw. (4993)  31 exp Sulfadoxine/ (2234)  32 sulfadoxine.tw. (2114)  33 exp Sulfadiazine/ (3777)  34 sulfadiazine.tw. (3361)  35 exp Clindamycin/ (5738)  36 clindamycin.tw. (10733)  37 exp Tetracyclines/ (48558)  38 tetracycline$.tw. (37372)  39 exp Minocycline/ (5978)  40 minocycline.tw. (6874)  41 exp Clarithromycin/ (6334)  42 clarithromycin.tw. (9352)  43 exp Azithromycin/ (5525)  44 azithromycin.tw. (8938)  45 exp Atovaquone/ (779)  46 atovaquone.tw. (1063)  47 exp Spiramycin/ (715)  48 spiramycin.tw. (1295)  49 exp Rifabutin/ (2126)  50 rifabutin.tw. (1249)  51 exp Trimetrexate/ (362)  52 trimetrexate.tw. (452)  53 exp Lincomycin/ (7528)  54 lincomycin.tw. (2620)  55 exp Dapsone/ (4862)  56 dapsone.tw. (4315)  57 exp Sulfisoxazole/ (948)  58 sulfafurazole.tw. (34)  59 sulfisoxazole.tw. (714)  60 exp Ciprofloxacin/ (13577)  61 ciprofloxacin.tw. (26603)  62 exp Doxycycline/ (9807)  63 doxycycline.tw. (13792)  64 exp Miocamycin/ (278)  65 mio?amycin.tw. (140)  66 exp Erythromycin/ (25562)  67 erythromycin.tw. (21271)  68 exp Macrolides/ (110934)  69 macrolide$.tw. (16435)  70 exp Sulfonamides/ (121751)  71 sulfonamide$.tw. (15553)  72 exp Sulfamerazine/ (473)  73 sulfamerazine.tw. (422)  74 exp Nifurtimox/ (456)  75 nifurtimox.tw. (792)  76 exp Methotrexate/ (38183)  77 methotrexate.tw. (41628)  78 23 or 24 or 25 or 26 or 27 or 28 or 29 or 30 or 31 or 32 or 33 or 34 or 35 or 36 or 37 or 38 or 39 or 40 or 41 or 42 or 43 or 44 or 45 or 46 or 47 or 48 or 49 or 50 or 51 or 52 or 53 or 54 or 55 or 56 or 57 or 58 or 59 or 60 or 61 or 62 or 63 or 64 or 65 or 66 or 67 or 68 or 69 or 70 or 71 or 72 or 73 or 74 or 75 or 76 or 77 (1823416)  79 randomized controlled trial.pt. (517599)  80 controlled clinical trial.pt. (93941)  81 random$.ab. (1141162)  82 placebo.ab. (212728)  83 clinical trials as topic.sh. (193721)  84 randomly.ab. (345339)  85 trial.ti. (229228)  86 79 or 80 or 81 or 82 or 83 or 84 or 85 (1600983)  87 exp animals/ not humans.sh. (4758490)  88 86 not 87 (1467088)  89 22 and 78 and 88 (50)  90 limit 89 to yr="2018 - 2021" (7) |
| **# of records identified** | 7 |

| **Search electronic report #2** | |
| --- | --- |
| **Search type** | Update |
| **Databases** | - Embase |
| **Platform** | Embase.com |
| **Search date** | 21/11/2020 |
| **Update date** | Undefined |
| **Range of search date** | 2018-2020 |
| **Language restrictions** | None |
| **Other limits** | RCT |
| **Search strategy (results)** | #1.'oculartoxoplasmosis'/exp358  #2.'toxoplasma'/exp20,175  #3.'toxoplasmosis'/exp26,293  #4.toxoplasm*:ab,ti33,031  #5.gondi*:ab,ti18,130  #6.'chorioretinitis'/exp4,183  #7.'retinitis'/exp30,850  #8.'choroiditis'/exp8,025  #9.'uveitis'/exp60,979  #10.retinochoroiditis:ab,ti766  #11.chorioretin*:ab,ti8,858  #12.uveitis:ab,ti25,554  #13.ocular:ab,ti163,534  #14.retinitis:ab,ti15,653  #15.choroidoretinitis:ab,ti22  #16.choroiditis:ab,ti1,434  #17.eye:ab,ti316,675  #18.ophthalmic:ab,ti45,262  #19.#2OR#3OR#4OR#540,546  #20.#6OR#7OR#8OR#9OR#10OR#11OR#12OR#13510,737  OR#14OR#15OR#16OR#17OR#18  #21.#19AND#204,274  #22.#1OR#214,297  #23.'antiinfectiveagent'/exp3,761,789  #24.'cotrimoxazole'/exp81,686  #25.'tmpsmx':ab,ti1,970  #26.(tmpNEAR/5smx):ab,ti2,219  #27.cotrimoxazole:ab,ti8,196  #28.trimethoprim*:ab,ti22,235  #29.'pyrimethamine'/exp10,395  #30.pyrimethamine:ab,ti5,942  #31.'sulfadoxine'/exp3,656  #32.sulfadoxine:ab,ti2,584  #33.'sulfadiazine'/exp9,057  #34.sulfadiazine:ab,ti4,003  #35.'clindamycin'/exp52,151  #36.clindamycin:ab,ti14,386  #37.'tetracyclinederivative'/exp179,503  #38.tetracycline*:ab,ti43,843  #39.'minocycline'/exp24,624  #40.minocycline:ab,ti9,368  #41.'clarithromycin'/exp37,492  #42.clarithromycin:ab,ti14,055  #43.'azithromycin'/exp38,081  #44.azithromycin:ab,ti13,883  #45.'atovaquone'/exp3,635  #46.atovaquone:ab,ti1,480  #47.'spiramycin'/exp4,523  #48.spiramycin:ab,ti1,471  #49.'rifabutin'/exp6,118  #50.rifabutin:ab,ti1,655  #51.'trimetrexate'/exp1,266  #52.trimetrexate:ab,ti497  #53.'lincomycin'/exp8,906  #54.lincomycin:ab,ti2,800  #55.'dapsone'/exp19,176  #56.dapsone:ab,ti6,165  #57.'sulfafurazole'/exp4,022  #58.sulfafurazole:ab,ti51  #59.sulfisoxazole:ab,ti797  #60.'ciprofloxacin'/exp101,283  #61.ciprofloxacin:ab,ti36,711  #62.'doxycycline'/exp54,085  #63.doxycycline:ab,ti20,210  #64.'miokamycin'/exp453  #65.mio?amycin:ab,ti174  #66.'erythromycin'/exp76,080  #67.erythromycin:ab,ti25,666  #68.'macrolide'/exp317,048  #69.macrolide*:ab,ti21,760  #70.'sulfonamide'/exp254,622  #71.sulfonamide*:ab,ti16,777  #72.'sulfamerazine'/exp1,506  #73.sulfamerazine:ab,ti457  #74.'nifurtimox'/exp1,979  #75.nifurtimox:ab,ti963  #76.'methotrexate'/exp182,728  #77.methotrexate:ab,ti68,820  #78.#23OR#24OR#25OR#26OR#27OR#28OR#29OR3,877,352  #30OR#31OR#32OR#33OR#34OR#35OR#36OR  #37OR#38OR#39OR#40OR#41OR#42OR#43OR  #44OR#45OR#46OR#47OR#48OR#49OR#50OR  #51OR#52OR#53OR#54OR#55OR#56OR#57OR  #58OR#59OR#60OR#61OR#62OR#63OR#64OR  #65OR#66OR#67OR#68OR#69OR#70OR#71OR  #72OR#73OR#74OR#75OR#76OR#77  #79.('randomizedcontrolledtrial'/expOR'single1,819,793  blindprocedure'/expOR'doubleblind  procedure'/expOR'crossoverprocedure'/expOR  random*:ab,tiORplacebo*:ab,tiORallocat*:ab,ti  ORcrossover*:ab,tiOR'crossover':ab,tiOR  trial:tiOR((doubl*NEXT/1blind*):ab,ti))NOT  (('animal'/deOR'animalexperiment'/deOR  'nonhuman'/de)NOT(('animal'/deOR'animal  experiment'/deOR'nonhuman'/de)AND'human'/de))  #80.#22AND#78AND#7962  #80.#22AND#78AND#79AND[embase]/lim56  #82.#22AND#78AND#79AND[embase]/limAND11[2018-2020]/py |
| **# of records identified** | 11 |

| **Search electronic report #3** | |
| --- | --- |
| **Search type** | Update |
| **Databases** | - EBM Reviews - Cochrane Central Register of Controlled Trials <October 2020> |
| **Platform** | Ovid.com |
| **Search date** | 21/11/2020 |
| **Update date** | Undefined |
| **Range of search date** | 2018-2020 |
| **Language restrictions** | None |
| **Other limits** | None |
| **Search strategy (results)** | 1 exp Toxoplasmosis, Ocular/ (17)  2 exp Toxoplasma/ (20)  3 exp Toxoplasmosis/ (91)  4 toxoplasm$.tw. (279)  5 gondi$.tw. (101)  6 exp Chorioretinitis/ (22)  7 exp Retinitis/ (181)  8 exp Choroiditis/ (48)  9 exp Uveitis/ (607)  10 retinochoroiditis.tw. (31)  11 chorioretin$.tw. (355)  12 uveitis.tw. (1130)  13 ocular.tw. (12543)  14 retinitis.tw. (444)  15 choroidoretinitis.tw. (0)  16 choroiditis.tw. (17)  17 eye.tw. (24365)  18 ophthalmic.tw. (4758)  19 2 or 3 or 4 or 5 (286)  20 6 or 7 or 8 or 9 or 10 or 11 or 12 or 13 or 14 or 15 or 16 or 17 or 18 (34347)  21 19 and 20 (64)  22 1 or 21 (65)  23 exp Anti-Infective Agents/ (68676)  24 exp Trimethoprim, Sulfamethoxazole Combination/ (1)  25 tmp-smx.tw. (230)  26 (tmp adj5 smx).tw. (254)  27 cotrimoxazole.tw. (475)  28 trimethoprim$.tw. (1517)  29 exp Pyrimethamine/ (716)  30 pyrimethamine.tw. (1233)  31 exp Sulfadoxine/ (586)  32 sulfadoxine.tw. (773)  33 exp Sulfadiazine/ (262)  34 sulfadiazine.tw. (371)  35 exp Clindamycin/ (870)  36 clindamycin.tw. (1627)  37 exp Tetracyclines/ (2520)  38 tetracycline$.tw. (1898)  39 exp Minocycline/ (528)  40 minocycline.tw. (990)  41 exp Clarithromycin/ (1463)  42 clarithromycin.tw. (3033)  43 exp Azithromycin/ (970)  44 azithromycin.tw. (2655)  45 exp Atovaquone/ (103)  46 atovaquone.tw. (166)  47 exp Spiramycin/ (28)  48 spiramycin.tw. (87)  49 exp Rifabutin/ (115)  50 rifabutin.tw. (218)  51 exp Trimetrexate/ (16)  52 trimetrexate.tw. (29)  53 exp Lincomycin/ (911)  54 lincomycin.tw. (68)  55 exp Dapsone/ (280)  56 dapsone.tw. (449)  57 exp Sulfisoxazole/ (69)  58 sulfafurazole.tw. (7)  59 sulfisoxazole.tw. (55)  60 exp Ciprofloxacin/ (1196)  61 ciprofloxacin.tw. (2418)  62 exp Doxycycline/ (1062)  63 doxycycline.tw. (1936)  64 exp Miocamycin/ (17)  65 mio?amycin.tw. (14)  66 exp Erythromycin/ (3348)  67 erythromycin.tw. (1805)  68 exp Macrolides/ (8755)  69 macrolide$.tw. (1084)  70 exp Sulfonamides/ (10267)  71 sulfonamide$.tw. (185)  72 exp Sulfamerazine/ (5)  73 sulfamerazine.tw. (1)  74 exp Nifurtimox/ (20)  75 nifurtimox.tw. (37)  76 exp Methotrexate/ (4132)  77 methotrexate.tw. (10015)  78 23 or 24 or 25 or 26 or 27 or 28 or 29 or 30 or 31 or 32 or 33 or 34 or 35 or 36 or 37 or 38 or 39 or 40 or 41 or 42 or 43 or 44 or 45 or 46 or 47 or 48 or 49 or 50 or 51 or 52 or 53 or 54 or 55 or 56 or 57 or 58 or 59 or 60 or 61 or 62 or 63 or 64 or 65 or 66 or 67 or 68 or 69 or 70 or 71 or 72 or 73 or 74 or 75 or 76 or 77 (96858)  79 22 and 78 (47)  80 limit 79 to yr="2018 -Current" (3) |
| **# of records identified** | 3 |

| **Search electronic report #4** | |
| --- | --- |
| **Search type** | Update |
| **Databases** | - Lilacs Via iAHx |
| **Platform** | Lilacs.bvsalud.org |
| **Search date** | 21/11/2020 |
| **Update date** | Undefined |
| **Range of search date** | 2018-2020 |
| **Language restrictions** | None |
| **Other limits** | Clinical Trials |
| **Search strategy (results)** | (mh:("Toxoplasmosis, Ocular")) OR (ti:(toxoplasmosis)) OR (ab:(toxoplasmosis)) AND db:("LILACS") AND type_of_study:("clinical_trials") AND ( db:("LILACS")) AND (year_cluster:[2018 TO 2020]) |
| **# of records identified** | 0 |

- **Total of records identified**

**MEDLINE + EMBASE + COCHRANE CENTRAL + LILACS = 7+11+3+0 = 21**

- **Number of records without duplicates = 12**

**In the update 2020, none of the studies evaluated in title and abstract met the selection criteria**
