# Supplementary material for: Safety and efficacy of different antibiotic regimens in patients with ocular toxoplasmosis: systematic review and meta-analysis
Source: Syst Rev. 2021 Jul 19;10:206. doi: 10.1186/s13643-021-01758-7 (PMC8287816; doi:10.1186/s13643-021-01758-7)
Supplement: Supplementary file 2 — Additional file 2. [file 13643_2021_1758_MOESM2_ESM.docx]

**ANNEX 2**

| **Intravitreal clindamycin compared to pyrimethamine sulfadiazine for ocular toxoplasmosis** | | | | | | | | | | | | |
| --- | --- | --- | --- | --- | --- | --- | --- | --- | --- | --- | --- | --- |
| **Patient or population:** patients with ocular toxoplasmosis **Intervention:** intravitreal clindamycin **Comparison:** pyrimethamine sulfadiazine | | | | | | | | | | | | |
| **Quality assessment** | | | | | | | **No of patients** | | **Effect** | | **Quality** | **Importance** |
|  |  |  |  |  |  |  |  |  |  |  |  |  |
| **No of studies** | **Design** | **Risk of bias** | **Inconsistency** | **Indirectness** | **Imprecision** | **Other considerations** | **Intravitreal clindamycin** | **Pyrimethamine sulfadiazine** | **Relative (95% CI)** | **Absolute** |  |  |
| **mean change in visual acuity (follow-up 6weeks to 6 months; measured with: logMAR; Better indicated by higher values)** | | | | | | | | | | | | |
| 2 | randomised trials | serious^1^ | no serious inconsistency | no serious indirectness | serious^2^ | none | 66 | 68 | - | MD 0.11 higher (0.03 to 0.20 higher) | ++oo LOW | CRITICAL |
| **Resolution of vitreous inflammation (follow-up 6 weeks to 6 months)** | | | | | | | | | | | | |
| 2 | randomized trials | serious^1^ | no serious inconsistency | no serious indirectness | very serious^3,4^ | none | 43/66  (65.2%) | 43/68  (63.2%) | RR 1.04 (0.83 to 1.31) | 25 more per 1000 (from 108 fewer to 196 more) | +ooo VERY LOW | CRITICAL |

^1^ High-risk performance and attrition bias
^2^ Only two randomized controlled trials contributed data. The overall treatment effect is precise with a narrow confidence interval [0.03 to 0.20] favoring pyrimethamine sulfadiazine. However, precision is rated down by one level due to the small sample size (n=134)
^3^ 95% confidence interval around the pooled or best estimate of effect includes both 1) no effect and 2) appreciable benefit or appreciable harm
^4^ Only two randomized controlled trials contributed data. Precision is rated down by one level due to the small sample size (n=134)

| **Trimethoprim + sulfamethoxazole compared to other antibiotics for ocular toxoplasmosis** | | | | | | | | | | | | |
| --- | --- | --- | --- | --- | --- | --- | --- | --- | --- | --- | --- | --- |
| **Patient or population: patients with ocular toxoplasmosis Intervention: Trimethoprim + sulfamethoxazole Comparison: other antibiotics (i.e pyrimethamine sulfadiazine or azithromycin)** | | | | | | | | | | | | |
| **Quality assessment** | | | | | | | **No of patients** | | **Effect** | | **Quality** | **Importance** |
|  |  |  |  |  |  |  |  |  |  |  |  |  |
| **No of studies** | **Design** | **Risk of bias** | **Inconsistency** | **Indirectness** | **Imprecision** | **Other considerations** | **Trimethoprim + sulfamethoxazole** | **Other antibiotics** | **Relative (95% CI)** | **Absolute** |  |  |
| **Resolution of vitreous inflammation (follow-up 6 to 12 weeks)** | | | | | | | | | | | | |
| 2 | randomized trials | serious^1^ | serious^2^ | no serious indirectness | very serious^3,4^ | none | 27/43  (62.8%) | 27/43  (62.8%) | RR 1.08 (0.59 to 1.98) | 50 more per 1000 (from 257 fewer to 615 more) | +ooo VERY LOW | CRITICAL |

^1^ High-risk performance and attrition bias
^2^ I2= 66%. Different antibiotics as a comparator (i.e. azithromycin and PYR/SDZ)
^3^ 95% confidence interval around the pooled or best estimate of effect includes both 1) no effect and 2) appreciable benefit or appreciable harm
^4^ Only two randomized controlled trials contributed data. Precision is rated down by one level due to the small sample size (n=88)
