# Supplementary material for: Safety and efficacy of different antibiotic regimens in patients with ocular toxoplasmosis: systematic review and meta-analysis
Source: Syst Rev. 2021 Jul 19;10:206. doi: 10.1186/s13643-021-01758-7 (PMC8287816; doi:10.1186/s13643-021-01758-7)
Supplement: Supplementary file 3 — Additional file 3. [file 13643_2021_1758_MOESM3_ESM.docx]

**ANNEX 3. Complete information on included studies**

***Balaskas 2012***

| **Methods** | Prospective, randomized, institutional pilot clinical study. | |
| --- | --- | --- |
| **Participants** | **Location:** Uveitis Clinic of the Jules Gonin Eye Hospital, Switzerland.  N=19. Intervention= 10, age =40,9 ± 17,1, range not reported, female 30% (n=3). Control= 9, age = 47,7 ± 19,1, range 20-42, female 55,6% (n=5). **Diagnosis:** Active, creamy-white focal retinal lesion, associated or not with an adjacent hyperpigmented chorioretinal scar. Serum anti-T. gondii IgG and IgM antibodies were determined in all patients and found to be consistent with the clinical diagnosis of active toxoplasma retinochoroiditis. | |
| **Interventions** | **Intervention:** azithromycin 500 mg qd + prednisone 1 mg/kg/day initiated 3 days after the beginning of antiparasitic treatment with a gradual tapering off by 5 mg every 3 days. **Control:** PYR 25 mg bid and SDZ 1000 mg tid for patients weighing less than 65 kg or 1000 mg qid for those weighing more than 65 kg + folinic acid 15 mg qd + prednisone 1 mg/kg/day initiated 3 days after the beginning of antiparasitic treatment with a gradual tapering off by 5 mg every 3 days. | |
| **Outcomes** | **Loss in follow up:** 0 %  **Changes in VA , Number of recurrences, Improvement in VA**  **Ocular inflammation (SUN), Lesion Size:** not reported  **Adverse drug reactions:** No data for intervention group; 10 minor adverse drug reactions in the control group (100%) but no severe reactions.  **Time of sharpening of lesion borders**  - Clinical evaluation (days): Intervention group 25,5 (IQR 23,00); Control group 4,0 (IQR 13,50); p=0.870  - Masked evaluation of photographs (days): Intervention group 30,5 (IQR 30,75); Control group 24,0 (IQR 17,00); p=0.270 **Time to lesion scarring** - Clinical evaluation (days): Intervention group 73,0 (IQR 57,25); Control group 47,0 (IQR 14,00); p=0.236  - Masked evaluation of photographs (days): Intervention group 71,5 (IQR 57,50) Control group 36,0 (IQR 25,50); p=0.307 **Time to disease inactivity (days)**  Intervention group 73,0 (IQR 61,25); Control group 49,0 (IQR 42,50); p=0.540  **Treatment tolerance (VAS score)**  Intervention group 8,5 (IQR 5,0); Control group 3,0 (IQR 4,0); p=0.0005 **Treatment failure**  Intervention group 1 (10%); Control group 0 (0%=); p>0.99 | |
| **Time Follow up** | Patients were examined on day 1 and then every 15±5 days until disease inactivity. A further visit at 3 months from the beginning of treatment was performed for all patients. | |
| **Source of funding / Conflict of interest** | Not reported / None of the authors have any financial or property interest  on any product. | |
| **Author's conclusion** | Azithromycin monotherapy at a dose of 500 mg per day was shown to be effective and well-tolerated for the treatment of active, non-vision-threatening toxoplasmic retinochoroiditis. The duration of treatment was clinically longer for the azithromycin group. | |
| **Risk of Bias** | | |
| **Bias** | **Author judgment** | **Support for judgment** |
| Randomization sequence generation (Selection bias) | Low risk | Random choice by the patient of a sealed envelope containing the treatment regimen |
| Assignment concealment (Selection bias) | Low risk | Idem |
| Masking of participants and staff (performance bias) | High risk | Unmasked |
| Masking the outcome assessment (detection bias) | Low risk | Fundus photographs were evaluated in a masked fashion by an independent ophthalmologist. |
| Incomplete data (Attrition bias) | Low risk | Complete data of all patients is available, there was no loss of follow-up. |
| Selective report (Report bias) | High risk | There is no protocol and not all the expected results are reported. |
| Other biases | Low risk | No other risk of bias was found. |

***Baharivand 2013***

| **Methods** | Prospective randomized single-blind clinical trial. | |
| --- | --- | --- |
| **Participants** | **Location:** Two referral teaching eye centers (Nikookari and Alavi), Iran.  N=66.  Intervention=32, age =25,69 ± 4,04, range 18-32, female 53,1% (n=17).  Control= 34, age = 27,21 ± 5,06, range 20-42, female 58,8% (n=20).  **Diagnosis:** clinical findings of retinal necrosis, retinitis, or vitritis in the absence of other identifiable causes, as well as positive serum titers of antibody (immunoglobulin Ig G or M) for T. gondii. | |
| **Interventions** | **Intervention**: intravitreal injection of 1 mg clindamycin + 400 picog dexamethasone  **Control:** PYR (initial dose of 75 mg/day for 2 days followed by 25 mg/day for 6 weeks), SDZ (initial dose of 2 g/day for 2 days followed by 1 g four times/day for 6 weeks), folinic acid (5 mg/day for 6 weeks), and oral prednisone (50 mg/day for 3 weeks starting from the third day of therapy). | |
| **Outcomes** | **Loss in follow-up %** : 3 in intervention group, 1 in control group  **Changes in Visual acuity:** Intervention group 0,38 ± 0,35 logMAR; Control group 0,35 ± 0,29 logMAR; p=0.31  **Number of recurrences:** 4 (12.5 %) in the clindamycin group and 5 (14.7 %) in control group; p = 0.54  **Improvement in visual acuity (gaining >=2 Snellen lines):** 27 out of 32 patients in intervention group and 28 out of 34 patients in control group; p = 0.83  **Ocular inflammation**  28 patients (87,5%) in intervention group and 28 patients (82,4%) in control group had grade 0 or trace inflammation, respectively; p=0.43  **Size of the lesion** **improvement:** 21 patients (65.6%) in intravitreal clindamycin group versus 23 patients (67.6%) in PYR/SDZ group, p=0.86  **Adverse drug reactions:** 1 in control group 2,9% with hepatotoxicity : The condition resolved after changing pyrimethamine– sulfadiazine to azithromycin | |
| **Time Follow up** | Patients were re-examined weekly for up to 6 months after the initiation of the treatments. | |
| **Source of funding / Conflict of interest** | Not reported / None | |
| **Author's conclusion** | Both intravitreal clindamycin + dexamethasone and PYR/SDZ are equally effective against active toxoplasma retinochoroiditis, but the former is safer and more convenient. | |
| **Risk of Bias** | | |
| **Bias** | **Author judgment** | **Support for judgment** |
| Randomization sequence generation (Selection bias) | Low risk | The participants were randomly allocated to two equal groups by a computer random number generation. |
| Assignment concealment (Selection bias) | Low risk | Randomization numbers were kept in a sealed document by a care provider not involved in the survey until the end of the study. |
| Masking of participants and staff (Performance bias) | High risk | There was no information about the masking of the patients and investigators. |
| Masking the outcome assessment (Detection bias) | Low risk | The outcome variables were evaluated by a masked skilled retina specialist at baseline and follow-ups. |
| Incomplete data (Attrition bias) | High risk | There was an imbalance in the losses during the study and the reasons for their loss are not explained. |
| Selective report (Report bias) | Low risk | Although there is no protocol all the expected results are reported. |
| Other biases | Low risk | No other risk of bias was found. |

***Bosch-Driessen 2002***

| **Methods** | Prospective, randomized open-labeled multicenter study. | |
| --- | --- | --- |
| **Participants** | **Location**: five ophthalmologic departments of the University Hospitals in The Netherlands.  N=46.  Intervention=24, age=not reported, range=not reported.  Control= 22, age=not reported, range=not reported.  **Diagnosis:** the presence of an active creamy-white colored focal retinal lesion with or without hyperpigmented retinochoroidal scars in either eye. When the initial diagnosis of ocular toxoplasmosis was uncertain, confirmation was obtained by intraocular fluid analysis. Serum IgG and IgM antibodies against T. gondii were determined and found to be in accord with the diagnosis of ocular toxoplasmosis in all patients. Sight-threatening ocular toxoplasmosis was considered in patients with macular location of lesions, lesions near the optic disk, or lesions larger than three optic disk diameters. | |
| **Interventions** | **Intervention:** PYR 100 mg on day 1 followed by 50 mg/day + azithromycin 250 mg/day or 500 mg every two days + folinic acid 15 mg/day + prednisone 40 mg from day 3 to day 10, followed by a gradual tapering off.  **Control:** PYR 100 mg on day 1 followed by 50 mg/day + sulfadiazine 4000 mg/day + folinic acid 15 mg/day + prednisone 40 mg from day 3 to day 10 followed by a gradual tapering off. | |
| **Outcomes** | **Loss in follow-up**: 3 patients from control group because of side effects  **Changes in Visual acuity:** Not reported  **Number of recurrences (at least 1 year follow up)**: Intervention group 5 /15 for interventenion group 33%); Control group 5 / 9 (56%); p = 0.26  **Improvement in visual acuity (>= 0.5 logMAR units at 3 months):** Intervention group 5 /24 (21%) patients; Control group 5/18 patients (28%); p=0.60  **Ocular inflammation** **(Disappearance of inflammatory cells from vitreous within 4 weeks):** Intervention group 14/20 (70%); Control group 10/14 (71%); p =0.60  **Size of the lesion improvement (decrease more than 0,5 optic disk diameter in 3 months):** Intervention group 9/22 (41 %); Control group 7/17 (41%); p=0.99.  **Adverse drug reactions (all):** Intervention group 8/24 (33%) for intervention group; Control group 14/22(64%); p=0.04 | |
| **Time Follow up** | Mean follow-up was 15 months in both groups (median, 12 months in the azithromycin group, and 11 months in the sulfadiazine group) | |
| **Source of funding / Conflict of interest** | Supported in part by the Dr. F.P. Fischer Foundation, The Netherlands / Not stated | |
| **Author's conclusion** | The efficacy of the multidrug regimen with pyrimethamine and azithromycin was like the standard treatment with pyrimethamine and sulfadiazine.  However, the frequency and severity of adverse drug reactions were significantly lower with a regimen containing pyrimethamine and azithromycin. | |
| **Risk of Bias** | | |
| **Bias** | **Author judgment** | **Support for judgment** |
| Randomization sequence generation (Selection bias) | Unclear | “Patients were assigned by randomization protocol” (It is not specified how). |
| Assignment concealment (Selection bias) | Unclear | It is not specified how. |
| Masking of participants and staff (Performance bias) | High | Open-label. |
| Masking the outcome assessment (Detection bias) | Unclear | Fundus photographs of the retinochoroidal lesions were evaluated by two independent ophthalmologists in a masked way. |
| Incomplete data (Attrition bias) | High risk | There is an imbalance in the reasons that caused the missing data. |
| Selective report (Report bias) | Unclear | The protocol is not available, and it is not clear if all expected results are reported. |
| Other biases | Unclear | It is not clear if there is an initial imbalance between the groups. |

***Colin 1989***

| **Methods** | Prospective randomized study. | |
| --- | --- | --- |
| **Participants** | **Location:** not exactly reported  N=29  Intervention=14, age=27.5, range=15-57, females=7  Control=15 age=24, range=9-64, females=7  **Diagnosis:** Ophthalmoscopic and angiographic appearance evocative of ocular toxoplasmosis (excluding chorioretinitis from other recognized causes) and which had a positive anti toxoplasma serology, even at a low rate. | |
| **Interventions** | **Intervention:** clindamycin was administered topically as subconjunctival injections at a unit dose of 50 mg. The protocol consisted of one injection per day for 5 days then 2 times per week for 3 weeks + oral prednisolone 1 to 1.5 mg (kg) for 1 to 2 weeks and a gradual decrease in dosage for 2 to 4 weeks.  **Control:** PYR 100 mg on the first day and then 50 mg daily for 6 + SDZ 4 g per day for 6 weeks + Intramuscular injection of 5 mg folinic acid) was performed every 4 days., oral prednisolone (1 to 1.5 mg (kg) for 1 to 2 weeks and a gradual decrease in dosage for 2 to 4 weeks. | |
| **Outcomes** | **Changes in VA, Improvement in VA, Ocular inflammation, Lesion Size:** Not reported  **Number of recurrences:** 21% in the intervention group, and 36% in the control group. (p-value not reported)  **Adverse drug reactions:** Intervention group one patient with corneal ulcer due to an error in the preparation of clindamycin; Control group one case of Stevens-Johnson syndrome.  **Visual acuity improvement was** measured subjectively, at 1 month of examination (100% patients in intervention group and 64% patients in the control group. | |
| **Time Follow up** | 14 months | |
| **Source of funding / Conflict of interest** | Not reported / not stated | |
| **Author's conclusion** | Subconjunctival injections of clindamycin provided an interesting alternative in the choice of anti-toxoplasma ocular therapy. | |
| **Risk of Bias** | | |
| **Bias** | **Author judgment** | **Support for judgment** |
| Randomization sequence generation (Selection bias) | Unclear | Insufficient information about the sequence generation process to permit judgment of ‘Low risk’ or ‘High risk’. |
| Assignment concealment (Selection bias) | Unclear | Insufficient information to permit judgment of ‘Low risk’ or ‘High risk’. |
| Masking of participants and staff (Performance bias) | High risk | No blinding or incomplete blinding, and the outcome is likely to be influenced by lack of blinding. |
| Masking the outcome assessment (Detection bias) | High risk | No blinding of outcome assessment and the outcome measurement is likely to be influenced by lack of blinding. |
| Incomplete data (Attrition bias) | Unclear | The study did not address this outcome. |
| Selective report (Report bias) | Unclear | Insufficient information to permit judgment of ‘Low risk’ or ‘High risk’. |
| Other biases | Unclear | Insufficient information to assess whether an important risk of bias exists. |

***Ghavidel 2017***

| **Methods** | Prospective randomized control clinical trial study. | |
| --- | --- | --- |
| **Participants** | **Location:** Outpatient uveitis clinic in Nikookari eye hospital, Tabriz, Iran.  N=72  Intervention= 36, age=41-94, range=27-56, female=18 (50%).  Control= 36, age=37.56, range=20-60, female=16 (44,6%).  **Diagnosis:** findings of visual disturbances and whitish-yellow appearing areas on the retina with a blurred margin matching focal chorioretinal necrotizing lesion with or without accompanying old lesion. | |
| **Interventions** | **Intervention:** Azithromycin 250 mg daily for 6 weeks + oral prednisolone 1 mg/kg daily starting 72 hours after initial therapy. The corticosteroid was tapered over 14 days.  **Control:** PYR/SDZ for 6 weeks + oral prednisolone 1 mg/kg daily starting 72 hours after initial therapy. The corticosteroid was tapered over 14 days. | |
| **Outcomes** | **Loss in follow-up:** None  **Changes in visual acuity**: Intervention group 0.35 logMAR (20/44 Snellen acuity); Control group 0.39 logMAR (20/49 Snellen acuity); p=0.33. Measures of dispersion not reported.  **Number of recurrences during 24 months after treatment:** Intervention group 18 (50%); Control group 4 (11.1 %); p= 0,00  **Improvement in Visual Acuity, Ocular Inflammation:** Not reported  **Retinal lesion size reduction** : Intervention group 354.86 micrometers; Control group 638.89 micrometers; p=0.098  **Adverse drug reactions (all):** Intervention group 4 (12,5%); Control group 20 (55,5%); p=0,00 | |
| **Time Follow up** | All patients were followed for 24 months during which they were examined by an ophthalmologist on the first day of treatment and then every 2 weeks until disease inactivity. Further visits were set every 3 months from the beginning of treatment for all participants. | |
| **Source of funding / Conflict of interest** | Not reported / None | |
| **Author's conclusion** | Azithromycin has shown to be effective for the treatment of active, non-vision threatening toxoplasma retinochoroiditis similar to classic treatment with PYR/SDZ regimen. However, recurrences and adverse drug reactions seem to differ significantly. | |
| **Risk of Bias** | | |
| **Bias** | **Author judgment** | **Support for judgment** |
| Randomization sequence generation (Selection bias) | Low Risk | Assigned to control or intervention arms of the study in a 1:1 ratio, utilizing a computer-generated randomization list. |
| Assignment concealment (Selection bias) | Unclear | The study mentioned to be a single-blind clinical trial, but there is no specification on how it was made. On how drugs were packed etc. |
| Masking of participants and staff (Performance bias) | High risk | Blinding of outcome assessment, but likely that the blinding could have been broken, and the outcome measurement is likely to be influenced by lack of blinding. |
| Masking the outcome assessment (Detection bias) | Low risk | No blinding of outcome assessment, but the review authors judge that the outcome measurement is not likely to be influenced by lack of blinding. |
| Incomplete data (Attrition bias) | Low Risk | No missing outcome data. |
| Selective report (Report bias) | Low Risk | The study protocol is not available, but the published reports include all expected outcomes, including those that were pre-specified (convincing text of this nature may be uncommon). |
| Other biases | Low Risk | The study appears to be free of other sources of bias. |

***Kartasasmita 2017***

| **Methods** | Randomized, single-blinded controlled trial study. | |
| --- | --- | --- |
| **Participants** | **Location:** Cicendo Eye Hospital, Bandung, Indonesia.  N=28.  Intervention=14, age =32.1, range=18-58.  Control=14, age=27.7, range=18-56.  **Diagnosis:** assessment of the morphology of the retinal lesions by a vitreoretinal specialist supported by positive IgG toxoplasma titer data. | |
| **Interventions** | **Intervention:** Clotrimoxazole 480 mg twice a day + oral clindamycin 300 mg four times daily for 3 weeks + oral methylprednisolone 1 mg/kg/d.  **Control:** PYR 25mg three times daily at the start of therapy, and then tapered to once daily + sulfadiazine 1000 mg twice daily at the beginning and then once daily + oral methylprednisolone 1 mg/kg/d tapered off each week + oral folic acid 5 mg that is given three times per week interval alternate days. The combination of drugs is given for 3 weeks. | |
| **Outcomes** | **Changes in VA, Number of recurrences, Improvement in VA**  **Ocular inflammation (SUN), Adverse drug reactions:** Not reported  **Retinal lesion size (percentage of lesion area reduction in third week)**: Intervention group 57.5%; Control group 52.5%; p=0.72 | |
| **Time Follow up** | They report data at the first and third week of follow-up | |
| **Source of funding / Conflict of interest** | Not reported / None | |
| **Author's conclusion** | Quadruple-drug therapy has a more rapid resolution effect on chorioretinitis lesions compared to triple therapy. | |
| **Risk of Bias** | | |
| **Bias** | **Author judgment** | **Support for judgment** |
| Randomization sequence generation (Selection bias) | Low risk | Random block permutation methods. |
| Assignment concealment (Selection bias) | Unclear risk | No information was provided. |
| Masking of participants and staff (Performance bias) | High risk | No blinding. |
| Masking the outcome assessment (Detection bias) | Low risk | The assessment result was made by calculating the retinal lesion using AutoCAD 2012, computer software by a blinded trained technician. |
| Incomplete data (Attrition bias) | Unclear risk | The study did not address this outcome. |
| Selective report (Report bias) | Unclear risk | Insufficient information to permit judgment of ‘Low risk’ or ‘High risk’. |
| Other biases | Low risk | The study appears to be free of other sources of bias. |

***Lashay 2017***

| **Methods** | Randomized interventional comparative study | |
| --- | --- | --- |
| **Participants** | **Location:** Retinal Department of Farabi Eye Hospital, Tehran, Iran.  N=27.  Intervention=14, age=24.9 ± 1.6, range=not reported, female=8 (57,2%).  Control=13, age=28 ± 1.5, range=not reported, females=5 (38,4%).  **Diagnosis:** the presence of an active white and bright focal retinal lesion with blurred margins with or without dark retinochoroidal scars. Confirmation was obtained by serum IgG and IgM antibodies against *T. gondii* in all patients. | |
| **Interventions** | **Intervention:** azithromycin 500 mg as a loading dose for one day followed by azithromycin 250 mg daily + oral prednisone 1 mg/ kg daily from the third day, and the dose was tapered over 2 weeks based on vitritis control.  **Control:** TMP 160mg + SMX 800 mg twice daily + oral prednisone 1 mg/ kg daily from the third day, and the dose was tapered over 2 weeks based on vitritis control. | |
| **Outcomes** | **Loss in follow-up:** none reported  **Changes in visual acuity** : Intervention group 0.24 ± 0.04 logMAR ; Control group 0.30 ± 0.01 logMAR; p=0,17  **Number of recurrences:** Not reported  **Improvement in VA:** Visual acuity improved in all cases after treatment except for 7 patients, 4 patients (14.8%) in intervention, and 3 patients (11.1%) in the control group  **Ocular inflammation** (vitreous inflammatory cells clearance): Intervention group 7/14 (50%); Control group 10/13 (77% ); p= 0.2  **Reduction in retinal size of lesion:** Intervention group 24.2 ± 6.5%; Control group 36.6 ± 4.6%; p=0.17  **Adverse drug reactions**: Intervention group 4 events (28,5%): 1 had skin irritation, 1 increase in serum bilirubin level to borderline values, and 2 patients mild diarrhea; Control group 3 events (23%): skin rashes in 1 patient, and fixed drug eruption in 3 patients (23%). | |
| **Time Follow up** | A masked ophthalmologist examined patients on day 1 and then after 1, 2, 3, 5, 6, 8, and 12 weeks. The patients were observed for at least 9 months after treatment completion. | |
| **Source of funding / Conflict of interest** | Not reported / None | |
| **Notes** | Drug efficacy in terms of reducing the size of retinal lesions and visual improvement was similar in a regimen of TMP/SMX or azithromycin treatment. Therefore, if confirmed with further studies, therapy with azithromycin seems to be an acceptable alternative for the treatment of ocular toxoplasmosis. | |
| **Risk of Bias** | | |
| **Bias** | **Author judgment** | **Support for judgment** |
| Randomization sequence generation (Selection bias) | Low Risk | Using block randomization. |
| Assignment concealment (Selection bias) | Unclear | Insufficient information to permit judgment of ‘Low risk’ or ‘High risk. |
| Masking of participants and staff (Performance bias) | High risk | No blinding or incomplete blinding, and the outcome is likely to be influenced by lack of blinding. |
| Masking the outcome assessment (Detection bias) | Low Risk | A masked ophthalmologist examined patients on day 1 and then after 1, 2, 3, 5, 6, 8, and 12 weeks. |
| Incomplete data (Attrition bias) | Unclear | Insufficient reporting of attrition/exclusions to permit judgment of ‘Low risk’ or ‘High risk’. |
| Selective report (Report bias) | Unclear | Insufficient information to permit judgment of ‘Low risk’ or ‘High risk’. |
| Other biases | Low Risk | The study appears to be free of other sources of bias. |

***Ortega 2000***

| **Methods** | Clinical controlled trial. | |
| --- | --- | --- |
| **Participants** | **Location:** Dr. Luis Sánchez Bulnes Hospital in Mexico  N=46  Intervention G1 =13, age=25,6, range=11-57, female=6 (46,1%)  Intervention G2=22, age=39,8, range=7-72, female=8 (36,3%)  Intervention G3=11, age=39,6, range=16-70, female=5 (45,4%) | |
| **Interventions** | **Intervention G1**: PYR 100 mg every 24 hours per two days and maintenance dose of 25mg every 24 hours + TMP/SMX 80 mg every 12 hours + 5 mg folinic acid every 24 hours v.o. for 8 weeks + prednisone 1 mg/kg/day one week and reduction in the following 7 weeks. It started 48 to 72 hrs after starting the antiparasitic.  **Intervention G2:** Clindamycin 300 mg every 6 hours v.o. for 8 weeks + TMP/SMX 80 mg every 12 hours + prednisone 1 mg/kg/day one week and reduction in the next 7 weeks.  **Intervention G3:** PYR 100 mg every 24 hours per two days and maintenance dose of 25mg every 24 hours + TMP/SMX 80 mg every 12 hours + clindamycin 300 mg every 6 hours v.o. for 8 weeks + 5 mg folinic acid every 24 hours for 8 weeks + prednisone 1 mg/kg/day one week and reduction in the following 7 weeks. | |
| **Outcomes** | **Loss in follow up, Changes in VA, Improvement in VA, Ocular inflammation, Lesion Size:** Not reported  **Number of recurrences :** Group 1 1(7.6%); Group 2 (31.2%); Group 3 (36.3%)  **Adverse drug reactions (called “Complications from treatment” and apparently mild):** Group 1: 1 (7.6%); Group 2: 4 (18.1%); Group 3: 0 (0%). **Gastrointestinal intolerance:** Group 1: 0; Group 2: 1 (4.5%); Group 3: 1 (9%). | |
| **Time Follow up** | Ophthalmological evaluation every 10/15 days. After the first two months, the patient was evaluated every 3-4 months or sooner, if he had ocular symptoms.  Group 1: 17 months  Group 2: 36.8 months  Group 3: 35.7 months | |
| **Source of funding / Conflict of interest** | Not reported / Not stated | |
| **Author's conclusion** | The final recommendation is the use of Pyrimethamine as the first-line antiparasitic and clindamycin as the second option. As the combination of these drugs does not offer additional benefits. | |
| **Risk of Bias** | | |
| **Bias** | **Author judgment** | **Support for judgment** |
| Randomization sequence generation (Selection bias) | High | The sequence generated by some rule was based on the date (or day) of admission. |
| Assignment concealment (Selection bias) | Unclear | Insufficient information to permit judgment of ‘Low risk’ or ‘High risk. |
| Masking of participants and staff (Performance bias) | High | No blinding or incomplete blinding, and the outcome is likely to be influenced by lack of blinding. |
| Masking the outcome assessment (Detection bias) | High | No blinding of outcome assessment and the outcome measurement is likely to be influenced by lack of blinding. |
| Incomplete data (Attrition bias) | High | Reason for missing outcome data likely to be related to true outcome, with either imbalance in numbers or reasons for missing data across intervention groups. |
| Selective report (Report bias) | Unclear | Insufficient information to permit judgment of ‘Low risk’ or ‘High risk’. |
| Other biases | High | They do not differentiate outcomes. |

***Soheilian 2005***

| **Methods** | Prospective randomized single-blind clinical trial. | |
| --- | --- | --- |
| **Participants** | **Location:** Labbafinejad Medical Center Uveitis Clinic in Tehran, Iran  N=59  Intervention=30, age=26.6 ± 11.7, range 12 to 59  Control=29, age=23.5 ± 7.4, range 12 to 45  **Diagnosis**: Patients clinically diagnosed with ocular toxoplasmosis, as defined by the presence of visual complaints and an area of focal necrotizing retinochoroidal lesion appearing as a whitish-yellow region with a blurred margin plus or minus the accompaniment of an old lesion. | |
| **Interventions** | **Intervention:** TMP 80 mg + SMX 400 mg every 12 hours + oral prednisolone, 1 mg/kg daily starting from the third day of therapy, and the dose was tapered over 2 weeks.  **Control:** PYR 100 mg for 2 days, followed by a 25-mg dose daily + SDZ 2 g daily for 2 days followed by 500-mg dosing every 6 hours + 5 mg of folinic acid daily + oral prednisolone 1 mg/kg daily starting from the third day of therapy, and the dose was tapered over 2 weeks. | |
| **Outcomes** | **Loss in follow up :** Intervention group 6 (16,7%): one for drug allergy and 5 for incomplete follow up; Control group 6 (17,1%): one due to development of allergic reaction to sulfadiazine and 5 due to incomplete follow-up  **Changes in Visual acuity :** increased by 0.56 logMAR units (5.5  lines) in the control group (*P*<0.01) and by 0.52 logMAR units (5 lines) in the intervention group (*P*<0.01); p=0.75  **Number of recurrences:** Intervention group: 3 (10%); Control group: 3 (10.3%); p=0.64  **Ocular inflammation** (Reduction of vitreous inflammatory cells (0–trace cells) 6 wks after treatment): Intervention group: 17 (56.7%) ; Control group: 20 (69%); p= 0.24  **Retinal lesion size (mean reduction after 6 weeks of treatment):** Intervention group: 59%; Control group 61%; *P* =0.75  **Adverse drug reactions:** Intervention group: 1 (2.8%); Control group: 1 (2.9%), rash in both cases; p= 0.98 | |
| **Time Follow up** | Patients were examined by an ophthalmologist on day 1, at the end of weeks 1 through 6, and every 3 months. Intervention group 31,5 ± 4,5 months Control group 33±4 months | |
| **Source of funding / Conflict of interest** | Supported in part by the Ophthalmic Research Center, Shahid Beheshti University of Medical Sciences, Tehran, Iran / None | |
| **Author's conclusion** | Drug efficacies in terms of reduction in retinal lesion size and improvement in VA were similar in a regimen of TMP/SMX and the classic treatment of ocular toxoplasmosis with PYR/SDZ. Therapy with TMP/SMX seems to be an acceptable alternative for the treatment of ocular toxoplasmosis. | |
| **Risk of Bias** | | |
| **Bias** | **Author judgment** | **Support for judgment** |
| Randomization sequence generation (Selection bias) | Low risk | Randomization tables were used. |
| Assignment concealment (Selection bias) | Unclear risk | No information. |
| Masking of participants and staff (Performance bias) | Low risk | To maintain patient masking, all medications were packaged similarly and labeled by number: 1 (the classic regimen) and 2 trimethoprim/sulfamethoxazole). Instruction for use was given by the same unmasked physician (M-MS). Unmasked ophthalmologists completed the physical examination, including VA measurement, slit-lamp examination, tonometry, funduscopy, and evaluation of laboratory tests, and then the patient consulted with a masked retina specialist for measuring the clinical outcomes of the study. |
| Masking the outcome assessment (Detection bias) | Low risk | Fundus photographs were evaluated by 2 independent masked observers. |
| Incomplete data (Attrition bias) | Unclear risk | Insufficient reporting of attrition/exclusions to permit judgment of ‘Low risk’ or ‘High risk’. |
| Selective report (Report bias) | Unclear risk | Insufficient information to permit judgment of ‘Low risk’ or ‘High risk’. |
| Other biases | Low risk | No other risk of bias. |

***Soheilian 2011***

| **Methods** | Controlled, randomized, single-blind clinical trial. | |
| --- | --- | --- |
| **Participants** | Location: Uveitis clinic of Labbafinejad Medical Center in Tehran  N=68  Intervention=34, age=24.5 ± 6.0, range not reported, female 18 (52,9%)  Control=34, age=23.3 ± 6.1, range not reported, female 16 (47,1%) | |
| **Interventions** | **Intervention:** Intravitreal injection of 1 mg clindamycin + 400 microg dexamethasone.  **Control:** PYR 25 mg daily (initial dose of 75 mg daily for 2 days) + SDZ 500 mg every 6 hours (initial dose of 4 g daily for 2days) + 5 mg folinic acid daily for 6 weeks + oral prednisolone 1 mg/kg daily for 3 weeks starting from the third day of therapy. | |
| **Outcomes** | **Loss in following-up:** Intervention group: 6 patients; Control group: 5 patients  **Changes in visual acuity** (% increase): Intervention group 0.44 ±0.24 logMAR; Control group 0.29±0.19 logMAR (74.4%); p= 0.174  **Number of recurrences:** 4 eyes (5.9%; 95% CI, 1.9%–13.6%; 2 in each group: 95% CI, 1%–18.1%) had 1 episode of recurrence. They occurred at 11 and 12 months in the intervention group and 8 and 13 months in the control group.  **Ocular inflammation (% patients with trace or no vitreous cells):** Intervention group 51.7% ; control group 55.5%; *P =* 0.563  **Retinal lesion size in pixels:** Intervention group: 116994 +- 143997 Pixels; Control group: 89606 +- 651553 pixels; p =0.571  **Retinal lesion size (mean % of changes):** Intervention group 58.4±29.3; Control group 57.0 ±27.6; p= 0.861  * Retinal lesion size was not measurable in 9 cases in the intervention group and 8 cases in the control group because of unacceptable quality of fundus photography  **Adverse drug reactions:** Intervention group: 4/34. Consisted in 3 subconjunctival hemorrhage (8.8%; 95% CI, 2.3%– 22.2%) and 1 transient raised intraocular pressure (2.9%; 95% CI, 0.1%–13.7%) that responded to medical therapy; Control group: 2/36 patients (5.6%; 95% CI, 0.9%–17.2%): skin rash in one and thrombocytopenia in the other. Both patients were excluded from the study. | |
| **Time Follow up** | All patients were examined weekly thereafter for up to 6 weeks. Patients were observed after completion of treatment for at least 24 months. | |
| **Source of funding / Conflict of interest** | Supported by Ophthalmic Research Center of Shahid Beheshti Medical  University, Tehran, Iran / None | |
| **Author's conclusion** | Intravitreal injection of clindamycin and dexamethasone may be an acceptable alternative to the classic treatment in ocular toxoplasmosis. It may offer the patient more convenience, a safer systemic side effect profile, greater availability, and fewer follow-up visits and hematologic evaluations. | |
| **Risk of Bias** | | |
| **Bias** | **Author judgment** | **Support for judgment** |
| Randomization sequence generation (Selection bias) | Low risk | Adverse drug reactions were limited to 2 of 36 patients in the CT group (5.6%; 95% CI, 0.9%–17.2%): skin rash in one and thrombocytopenia in the other (2.8%; 95% CI, 0.1%–12.9%). Both patients were discontinued from their medication regimens and were excluded from the study. |
| Assignment concealment (Selection bias) | Low risk | Performed by a biostatistician, putting the group sequence (written on the card) in envelopes labeled by ordered number. |
| Masking of participants and staff (Performance bias) | High risk | No blinding or incomplete blinding, and the outcome is likely to be influenced by lack of blinding. |
| Masking the outcome assessment (Detection bias) | Low Risk | A masked retina specialist. |
| Incomplete data (Attrition bias) | High risk | Imbalance in reasons for missing data across intervention groups. |
| Selective report (Report bias) | Low risk | The study protocol is not available, but the published reports include all expected outcomes, including those that were pre-specified (convincing text of this nature may be uncommon). |
| Other biases | Low risk | The study appears to be free of other sources of bias. |
