# Supplementary material for: Safety and efficacy of different antibiotic regimens in patients with ocular toxoplasmosis: systematic review and meta-analysis
Source: Syst Rev. 2021 Jul 19;10:206. doi: 10.1186/s13643-021-01758-7 (PMC8287816; doi:10.1186/s13643-021-01758-7)
Supplement: Supplementary file 4 — Additional file 4. [file 13643_2021_1758_MOESM4_ESM.docx]

**ANNEX 4**

| **AMSTAR 2.0 ASSESSMENT** | **FELICIANO ET AL 2020** | **ARTICLE SECTION** | **ZHANG ET AL 2018** | **ARTICLE SECTION** |
| --- | --- | --- | --- | --- |
| **1. Did the research questions and inclusion criteria for the review include the components of PICO?** | **YES** | **Selection criteria are found within the**  **Methods´ section**  *“****Participants*** *included were patients of any age who received antibiotic treatment for acute OT worldwide, as well as those with healed scars who received prophylactic antibiotic treatment to prevent recurrent or new lesions, including immunocompetent patients, immunosuppressed patients, pregnant women, and children”*  ***Intervention and comparison:*** *“comparison of therapies that have been used in OT at any dosage, duration, and administration route oral or intravitreal, relative to another antibiotic regimen” … including “prophylactic antibiotic treatment”*  ***Outcome****: “Primary outcome measures were changed in visual acuity at least three months after the start of treatment and the number of recurrences at the end of follow-up. Secondary outcome measures included the behavior of ocular inflammation signs according to the Standardization of Uveitis Nomenclature (SUN), size of lesion at the end of the follow-up, adverse events, and duration of the active lesion, as stated in the study protocol”* | **YES** | **Abstract and study outcomes subheading**  ***“****… OT in immunocompetent patients…The included studies were performed to evaluate the various treatment modalities of OT. Different treatment regimens were compared with …The outcomes relevant to the efficacy were as follows: (1) numbers of patients having an improvement of visual acuity or change in visual acuity compared with the baseline; (2) numbers of patients having recurrent retinochoroiditis at the end of the follow-up duration; (3) numbers of patients reporting a reduction of vitreous inflammation. The outcome relevant to the tolerability was presented by the number of patients reporting any adverse effect.* ***“*** |
| **2. Did the report of the review contain an explicit statement that the review methods were established prior to conduct of the review and did the report justify any significant deviations from the protocol?** | **YES** | **Methods´ section / Discussion**  **The protocol was previously published**  *“Antibiotic treatment for ocular*  *toxoplasmosis: a systematic review and*  *meta-analysis: study protocol“* [*https://doi.org/10.1186/s13643-019-1067-*](https://doi.org/10.1186/s13643-019-1067-)  *“This study protocol was developed based on the Preferred Reporting Items for Systematic Reviews and Meta-Analysis Protocols (PRISMA-P) guidelines [20]. It was published by Feliciano-Alfonso et al., Systematic Reviews 2019 [21]. In the same way, it was registered in PROSPERO, with the registration number CRD42018085468*“  “*We made changes regarding the published protocol as most primary outcomes established in the study protocol were not settled in the articles found by the search strategy”* | **NO** | **Information not provided in the article** |
| **3. Did the review authors explain their selection of the study designs for inclusion in the review?** | **YES** | **Included in Methods´ section**  *“We included all published and unpublished randomized controlled trials (RCTs) because well-conducted RCTs provide the highest quality of evidence according to the grading of recommendation assessment, development, and evaluation (GRADE) classification”* | **YES** | **Including in Methods´ section**  *“Randomized and quasi-randomized controlled trials that fulfilled the criteria were included. References of all randomized clinical trials were scanned for additional eligible article. ”* |
| **4. Did the review authors use a comprehensive literature search strategy?** | **YES** | **Included in Methods´ section and also found in Annex 1**  *“We used a combination of exploded controlled vocabulary with thesaurus Science Health Descriptors (DeCS for its Spanish acronym), Medical Subject Heading (MeSH) and Embase Subject Headings (Emtree) and free-text terms (considering spelling variants, plurals, synonyms, acronyms, and abbreviations) with field labels, truncation, proximity operators, and Boolean*  *operators. We conducted our search in the MEDLINE, EMBASE, Cochrane Central Register of Controlled Trials, and LILACS electronic databases, from inception to March 2018 (Annex 1). Also, the search was updated in November 2020. For identification of additional studies, the following resources were used: WHO International Clinical Trials Registry Platform portal, ClinicalTrials.gov, Gray Literature in Europe (“OpenGrey”), and manual searches within reference lists of all relevant studies identified by other methods.”*  *“We contacted study investigators to obtain missing data”.*  *“the search was updated in February 2020”* | **PARTIAL YES** | **Including in Methods´ section**  **There is no information of searching in gray literature nor clinical trials registries** |
| **5. Did the review authors perform study selection in duplicate?** | **YES** | **Included in Methods´ section**  *“For studies obtained by electronic searches, two review authors (JFA and AVV) independently reviewed titles and abstracts of all studies, then retrieved potentially relevant studies in PDF format. They also compared the PDF files with the inclusion criteria. Disagreements were resolved by consensus or by independent evaluation by a third review author (ADLT).”* | **YES** | **Including in Methods´ section**  *“Two authors independently inspected each reference that fulfilled the inclusion criteria and separately extracted the data on authors, year of publication, study design, baseline information, intervention, comparison, dosage, visual acuity, vitreous inflammation, recurrence, and side-effects.”* |
| **6. Did the review authors perform data extraction in duplicate?** | **YES** | **Included in Methods´ section**  *“A data collection form was designed. Then, two review authors (JMO and JFA) independently extracted relevant details regarding the design and results of each study. Disagreements were resolved by consensus or by independent evaluation by a third review author (ADLT)”* | **YES** | **Including in Methods´ section**  *“Two authors independently inspected each reference that fulfilled the inclusion criteria and separately extracted the data on authors, year of publication, study design, baseline information,intervention, comparison, dosage, visual acuity, vitreous inflammation, recurrence, and side-effects.”* |
| **7. Did the review authors provide a list of excluded studies** | **YES** | **Included in Results´ section, in Figure 1, and in the reference section.**  *“Five studies were excluded for reasons indicated in Figure 1 [29–34].”* | **NO** | **Despite in figure 1 was mentioned the exclusion of 47 articles, a list of all potentially relevant studies that were read in full-text form but excluded from the review was not provided** |
| **8. Did the review authors describe the included studies in adequate detail?** | **YES** | **Included in Results´ section (Characteristics of included studies Subheading) and in Annex 3 (Characteristics of included studies).** | **PARTIAL YES** | **Including in the Results section, and in Table 1.**  **Table 1 describes all the included studies, yet research designs and study´s setting is not included, therefore not fulfilling all required criteria for grading a “YES”**  *“…Ultimately, 10 trials were included, involving a total of 748 patients. Withdrawals or drop-outs were reported in 60% (6/10) of the trials. None of the conference abstracts were included for not fulfilling the inclusion criteria. We identified 10 randomized controlled trials, which were compared clindamycin, azithromycin, and TMP-SMX with conventional therapy (P-S plus glucocorticoids) or placebo.”* |
| **9. Did the review authors use a satisfactory technique for assessing the risk of bias (RoB) in individual studies that were included**  **in the review?** | **YES** | **Including in Discussion section 4.2 Potential biases in the review process**  *“We aimed to minimize potential biases by conducting a highly sensitive search for trials; we followed rigorous methods as recommended by the Cochrane group “Risk of Bias” tool for RCTs and criteria in the Cochrane Handbook for Systematic Reviews of Interventions. There were some limitations in our study, as it was not a network meta-analysis, such as the study published by Zhang et al ...”* | **NO** | **Information Not provided in the article** |
| **10. Did the review authors report on the sources of funding for the studies included in the review?** | **YES** | **Annex 3 (Characteristics of included studies).** | **NO** | **Information not provided in the article** |
| **11. If meta-analysis was justified did the review authors use appropriate methods for statistical combination of results?** | **YES** | **Including in Methods´ section**  *“We used a random-effects model to calculate our meta-analysis if eligible studies represented clinically varied populations. We used a fixed-effect meta-analysis for combining data where it was reasonable to assume that studies estimated the same underlying treatment effect.”* | **YES** | **Data analysis and interpretation**  *“All statistical analyses were performed using R version 3.2.5. Rank-probability test of network meta-analysis was created using pcnetmeta R package applied to the dataset illustrating probabilities that each treatment was ranked 1st–5th.”* |
| **12. If meta-analysis was performed did the review authors assess the potential impact of RoB in individual studies on the results of the meta-analysis or other evidence synthesis?** | **YES** | **Including in Results´ section**  *“The bias risk assessments for all included studies are summarized in Figures 2 and 3.”* | **NO** | **Information not provided in the article** |
| **13. Did the review authors account for RoB in individual studies when interpreting/ discussing the results of the review?** | **YES** | **Including in Results´ section**  *Yes, it was one of the items evaluated in GRADE methodology Annex 2. Also, it is discussed in section 4.1 Agreements and disagreements with other studies or reviews* | **NO** | **Information not provided in the article** |
| **14. Did the review authors provide a satisfactory explanation for, and discussion of, any heterogeneity observed in the results of the review?** | **YES** | **Including in Results´ section /Effects of interventions subheading**  *Yes, it was one of the items evaluated in GRADE methodology Annex 2 (Item inconsistency).* | **NO** | **Information not provided in the article** |
| **15. If they performed quantitative synthesis did the review authors carry out an adequate investigation of publication bias (small study bias) and discuss its likely impact on the results of the review?** | **YES** | **Including in Results´ section 3.3**  *“It was not possible to assess the publication bias since the number of studies necessary to evaluate it was not enough.”* | **NO** | **Information not provided in the article** |
| **16. Did the review authors report any potential sources of conflict of interest, including any funding they received for conducting the review?** | **YES** | **7.3 Competing interest and 7.4 Funding section**  *“The authors declare that they have no competing interests … This research did not receive any specific grant from funding agencies in the public, commercial, or not-for-profit sectors”.* | **YES** | **Acknowledgments section**  *“Research reported in this publication was supported in part by the Natural Science Foundation of China (no.81471973); the 2016 Medical Education Research Project of Chinese Medical Association Medical Education Branch and China Higher Education Society of Medical Education Professional Committee (2016B-KY013), and the Education and Teaching Reform Project of Sun Yat-sen University, China (2016)”.* |
| **Rating overall confidence in the results of the review** | **High**  No or one non-critical weakness: the systematic review provides an accurate and comprehensive summary of the results of the available studies that address the question of interest | | **Critically low** More than one critical flaw (Domains 2, 7, 9, 13, and 15) with or without non-critical weaknesses: the review has more than one critical flaw and should not be relied on to provide an accurate and comprehensive summary of the available studies | |
